# Association of Peripheral Blood Levels of Cytokines With Autism Spectrum Disorder: A Meta-Analysis

**DOI:** 10.3389/fpsyt.2021.670200

**Published:** 2021-07-02

**Authors:** Huaying Zhao, Hongqi Zhang, Shijie Liu, Wulin Luo, Yongfeng Jiang, Junwei Gao

**Affiliations:** ^1^Department of Rehabilitation Medicine, Fuling Central Hospital of Chongqing City, Chongqing, China; ^2^Department of Pulmonary and Critical Care Medicine, Fuling Central Hospital of Chongqing City, Chongqing, China; ^3^The 947th Hospital of Army, Kashi, China; ^4^Department of Medical Psychology and Neurology, The 947th Hospital of Army, Kashi, China; ^5^Department of Rehabilitation Medicine, The 947th Hospital of Army, Kashi, China; ^6^Department of Military Cognitive Psychology, School of Psychology, Third Military Medical University (Army Medical University), Chongqing, China

**Keywords:** cytokines, autism spectrum disorder, association, serum, plasma

## Abstract

**Background:** Although increasing evidence suggests an association between alterations in peripheral cytokines and autism spectrum disorder (ASD), a consensus is lacking. To determine whether abnormal cytokine profiles in peripheral blood were associated with ASD, we performed this systemic review and meta-analysis.

**Methods:** A systematic literature search was conducted through the Embase, PubMed, Web of Knowledge, PsycINFO, and Cochrane databases up to 4 June 2020. Clinical studies exploring the aberration of peripheral cytokines of autistic patients and controls were included in our meta-analysis. We pooled extracted data using fixed- or random-effects models based on heterogeneity tests with Comprehensive Meta-analysis software. We converted standardized mean differences to Hedges' g statistic to obtain the effect sizes adjusted for sample size. Subgroup analyses, sensitivity analyses, meta-regression, and publication bias tests were also carried out.

**Results:** Sixty-one articles (326 studies) were included to assess the association between 76 cytokines and ASD. We conducted our meta-analysis based on 37 cytokines with 289 studies. Since there were fewer than three studies on any of the other 39 cytokines, we only provided basic information for them. The levels of peripheral IL-6, IL-1β, IL-12p70, macrophage migration inhibitory factor (MIF), eotaxin-1, monocyte chemotactic protein-1 (MCP-1), IL-8, IL-7, IL-2, IL-12, tumor necrosis factor-α (TNF-α), IL-17, and IL-4 were defined as abnormal cytokines in the peripheral blood of ASD patients compared with controls. The other 24 cytokines did not obviously change in ASD patients compared with the controls.

**Conclusions:** The findings of our meta-analysis strengthen the evidence for an abnormal cytokine profile in ASD. These abnormal cytokines may be potential biomarkers for the diagnosis and treatment of ASD in the future.

## Background

Autism spectrum disorders (ASDs) are a group of neurodevelopmental disorders classically characterized by impaired social communication, restricted, repetitive patterns of behavior, and selective attention (1). Recent surveys indicate that ~1.5% of children and 0.8% of adolescents in the USA and UK are diagnosed with ASD (2). Although some investigations have suggested that genetic and environmental factors may affect ASD development, the biological mechanisms of these disorders remain poorly understood, and there are no specific medicines for ASD (3). Consequently, there is a pressing need to better understand the etiology of ASD and further develop appropriate intervention techniques and drugs.

The immune system is classically divided into two main branches: adaptive and innate immunity. Growing evidence suggests that the immune system plays a key role in ASD development (4). Adaptive immune cells, such as B cells and T cells, can develop antigen-specific responses, and give rise to immunological memory in the central nervous system (CNS) (5). In different parts of the brain of autistic patients, the increase in lymphocytes and immune dysfunction may lead to astrocyte damage, affecting the CSF-brain barrier, and contributing to the development of ASD (6). Innate immune cells, such as microglia and monocytes, can recognize conserved structures expressed by pathogens in the central nervous system. Abnormal synaptic pruning caused by microglia may affect synaptic plasticity and result in ASD (7). In addition, a series of inflammatory reactions involving microglia and cytokines are closely related to ASD (8). Cytokines, as a group of cell-signaling molecules, play vital roles in the regulation of the host immune response, and altered expression of cytokines is associated with ASD development (9). We usually classify cytokines into three groups according to the type of immune response: adaptive immunity, proinflammatory signaling, and anti-inflammatory signaling (10, 11). Cytokines, such as interleukin-6 (IL-6) and tumor necrosis factor-α (TNF-α), mediate communication between the immune system and brain (12). Animal studies have indicated an association between cytokines and psychiatric disorders, such as depression, schizophrenia, and ASD (13–15). Moreover, abnormal cytokine profiles were found in ASD patients compared to healthy controls in brain biopsies (16–18). On the other hand, peripheral cytokines can cross the blood-brain barrier and signal to the brain, which plays important roles in the development of the brain. Therefore, comprehensive analysis of the levels of peripheral cytokines may provide a new approach to improve the prognosis of autistic patients.

Several meta-analyses have been performed to review the associations between peripheral cytokine levels and ASD (19–21). However, some chemokines were not included or some data were included repeatedly. Moreover, in the past decade, an increasing number of studies have been conducted to detect the peripheral blood levels of different cytokines in autistic patients compared with controls. Considering the above content, we carried out a systemic review and meta-analysis.

## Methods

### Search Strategy and Study Selection

Two independent investigators (JG and HZ) conducted a systematic search of peer-reviewed records in the Embase, PubMed, Web of Knowledge, PsycINFO and Cochrane databases up to 4 June 2020 to identify the relevant studies. The keywords in our strategy were as follows: “cytokine [All Fields],” “chemokine [All Fields],” “interleukin [All Fields],” “interferon [All Fields],” “inflammation [All Fields],” or “tumor necrosis factor [All Fields];” AND “autism [All Fields],” “autistic [All Fields],” or “ASD [All Fields].” The combination of search keywords was used in all five databases. In addition, we checked the references according to the retrieved studies. Authors were also contacted to obtain original data not revealed in the original human articles, which could maximize the sample size.

The records retrieved were screened according to the inclusion and exclusion criteria. In our meta-analysis, the inclusion criteria were as follows: (1) original human articles; (2) clinical studies exploring the aberration of cytokines in autistic patients and controls; (3) cytokines detected in plasma or serum; (4) each cytokine concentration was presented as a mean with corresponding standard deviation (SD), or sample size and *P*-value. In addition, some concentrations were calculated based on the original data. The exclusion criteria were as follows: (1) studies with insufficient information such as concentration or *P*-value; (2) animal study, review, comment or abstract; (3) cytokines determined in CSF or other tissues; (4) studies reported levels of cytokines after cell stimulation tests. If some studies contained overlapping populations, only the study with the largest sample size was included. We did not estimate some data based on graphical figures or other formats (median, interquartile range) for the unwanted error. Our meta-analysis followed the guidelines recommended by the Preferred Reporting Items for Systematic Reviews and Meta-analysis (the PRISMA statement).

### Data Extraction

Two investigators (JG and HZ) independently screened the full text of the articles and extracted useful data to reduce personal error. Useful data were collected as follows: first author's name and publication year, country, sample type (serum or plasma), detection method, mean and SD of each cytokine in cases and controls, or *P*-value from the original studies. When a *P*-value was reported as an inequality rather than an exact value, we performed two extreme processing of the *P*-value to decrease type II errors. Pre-designed standard data forms were used for the information collected. Disagreement was resolved by discussion.

### Statistical Analysis

We calculated the effect sizes (ESs) as standardized mean differences (SMDs) in different levels of cytokines between cases and controls according to the extracted sample size, mean, and SD, or sample size and *P*-value when the mean and SD were unavailable. Then, we converted these SMDs to Hedges' g statistic to obtain the effect sizes adjusted for sample size. ESs and their 95% confidence intervals (CIs) were used to assess the association between different peripheral levels of cytokines and ASD. Heterogeneity across studies was evaluated with the *I*^2^ value and Cochrane *Q*-test. We selected a random-effects model in our meta-analysis when obvious heterogeneity was detected across studies; otherwise, a fixed-effects model was used. Publication bias was examined by Egger's test qualitatively (the more symmetrical, the lower risk of publication bias) and quantitatively (*P*-value).

Due to the limitation of the number of studies, the source of heterogeneity was only discussed in cytokines with a number of more than 10 studies. We conducted unrestricted maximum likelihood random-effects meta-regressions of ESs to verify whether some theoretical covariates, such as age and sample size, would serve as confounders to affect our results. Moreover, Galbraith plots were also employed to further investigate the source of heterogeneity across studies. Sensitivity analysis was performed for statistically significant ES estimates by sequentially removing individual studies to determine whether a single study could turn the relevance of results or otherwise change the direction of ES in our study. Comprehensive Meta-analysis software 2.0 and Stata 15.0 were selected to carry out all the statistical analyses in our study. *P* < 0.05 was considered significantly different in our study.

## Results

### Study Characteristics

**Figure 1:**
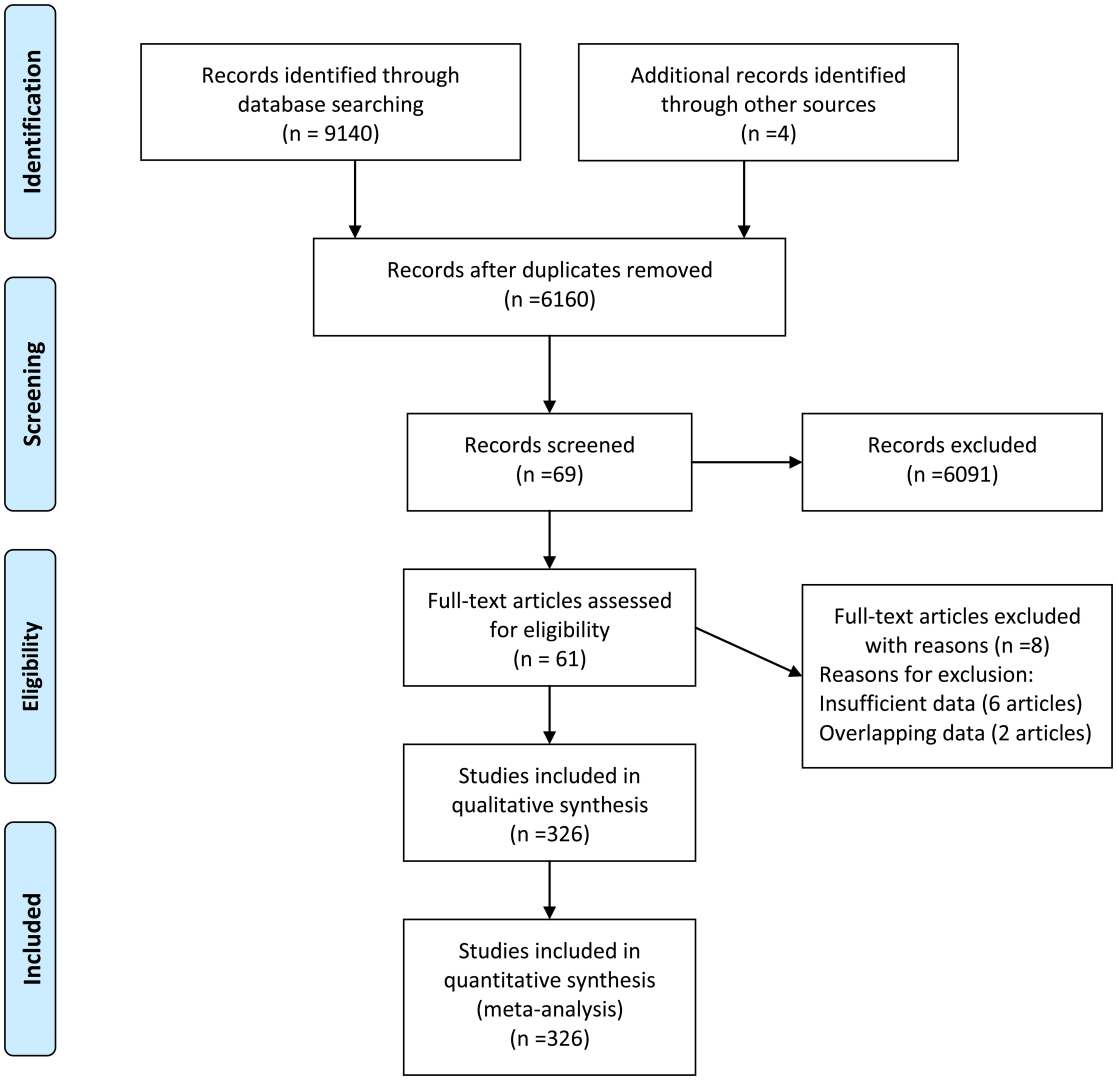
Flow diagram of study identification.

There were 9,144 records identified in the databases (2,805 from Embase, 1,457 from PubMed, 2,163 from Web of Knowledge, 2,618 PsycINFO, and 97 from Cochrane databases) updated to June 4, 2020, and four records were identified from the references. There were 2,984 duplications and 6,091 records with unmatched titles or abstracts that were excluded from the retrieved results. In addition, eight records were removed from the last 69 records by full text reading (six records with insufficient data and two with overlapping data). Then, 61 records were eligible for the next qualitative synthesis (22–82). When a record reported different cytokines, we treated them as separate studies. Therefore, a total of 326 studies were identified in our meta-analysis. The study selection process is shown in Figure 1.

There were 76 cytokines included in our study, 37 of which were analyzed based on extracted data. The diagnosis of ASD in most studies was made according to the Diagnostic and Statistical Manual of Mental Disorders. Most controls were healthy children. The concentrations of all cytokines were measured either in plasma or serum. Since some records contained multiple studies and the sample size of each study was different, we only exhibited the largest sample size. Eight cytokines (IL-6, IFNγ, IL-8, TNF-α, IL-1β, IL-10, IL-17, and IL-4) were detected in more than nine studies. Details are shown in Supplementary Table 1. In addition, we also assessed the association between the concentrations of some chemokines (such as eotaxin, MIP-1α, and Mig) and ASD.

The other 39 cytokines presented some basic characteristics for fewer than three studies. Eleven cytokines were detected in the two studies while 28 cytokines were measured in one study. Most of the 50 studies suggested no obvious association between the concentrations of these cytokines and ASD. More studies are required to further confirm the results. Details are shown in Table 1.

**Table 1:** Summary of studies on the unanalyzed cytokines.

**Cytokine**	**Sample type**	**Sample size (*****n*****)**		**Results**	**References**
		**Case**	**Control**		
IL-6sR	Plasma	25	25	NA	(48)
Eotaxin-2	Plasma	25	25	NA	(48)
Eotaxin-3	Serum	30	29	The concentration of Eotaxin-3 in ASD group was significantly decreased	(54)
Fractalkine	Plasma	87	41	NA	(78)
	Plasma	99	40	The concentration of Fractalkine in ASD group was significantly decreased	(44)
IFNα2	Plasma	28	28	NA	(40)
IL-1	Serum	77	77	The concentration of IL-1 in ASD group was significantly increased	(27)
	Serum	23	33	NA	(22)
MCP-3	Plasma	99	40	The concentration of MCP-3 in ASD group was significantly decreased	(44)
	Plasma	28	28	NA	(40)
MIP-1δ	Plasma	25	25	NA	(48)
TNF sRI	Plasma	25	25	NA	(48)
TNF sRII	Plasma	25	25	NA	(48)
EGFR	Plasma	33	34	The concentration of EGFR in ASD group was significantly increased	(53)
HB-EGF	Serum	30	29	The concentration of HB-EGF in ASD group was significantly decreased	(54)
NGF	Plasma	21	16	The concentration of NGF in ASD group was significantly increased	(77)
	Plasma	28	28	NA	(40)
IL-17F	Serum	32	28	NA	(76)
IL-22	Serum	32	28	NA	(76)
IL-21	Serum	32	28	NA	(76)
GRO-α	Serum	65	30	NA	(72)
	Plasma	28	28	The concentration of GRO-α in ASD group was significantly increased	(40)
Basic FGF	Serum	12	8	NA	(69)
M-CSF	Plasma	25	25	NA	(48)
	Plasma	28	28	NA	(40)
PDGF-AA	Serum	31	31	NA	(36)
PDGF-AB	Serum	31	31	NA	(36)
ENA-78	Serum	62	62	The concentration of ENA-78 in ASD group was significantly increased	(59)
	Serum	21	15	NA	(41)
IL-33	Plasma	30	18	NA	(56)
	Serum	38	13	NA	(61)
ST2	Plasma	30	18	NA	(56)
NT-3	Plasma	24	24	The concentration of NT-3 in ASD group was significantly decreased	(45)
BLC	Plasma	25	25	NA	(48)
I-309	Plasma	25	25	NA	(48)
IL-11	Plasma	25	25	NA	(48)
IL-16	Plasma	25	25	NA	(48)
	Plasma	28	28	NA	(40)
MDC	Serum	56	32	The concentration of MDC in ASD group was significantly increased	(46)
	Serum	37	37	The concentration of MDC in ASD group was significantly decreased	(49)
TARC	Serum	56	32	The concentration of TARC in ASD group was significantly increased	(46)
IL-3	Serum	37	37	The concentration of IL-3 in ASD group was significantly decreased	(49)
	Plasma	28	28	NA	(40)
I-TAC	Serum	21	15	NA	(41)
CTACK	Plasma	28	28	NA	(40)
SCF	Plasma	28	28	NA	(40)
SCGF-β	Plasma	28	28	NA	(40)
SDF-1α	Plasma	28	28	NA	(40)
TRAIL	Plasma	28	28	NA	(40)
IL-31	Serum	38	13	NA	(55)

*NA, not association*.

### Quantitative Data Synthesis

**Figure 2:**
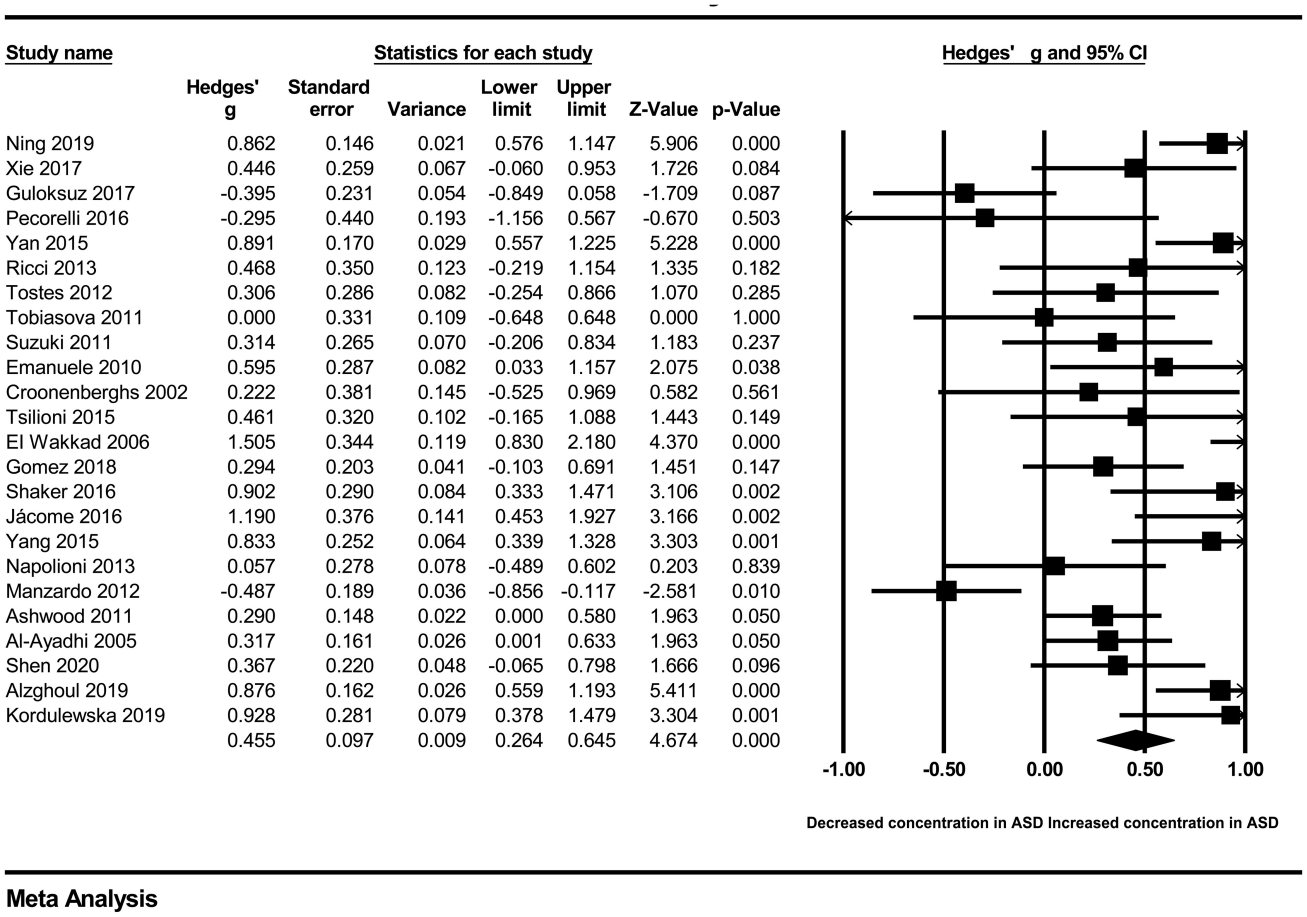
Forest plot for the random-effects meta-analysis (IL-6).

**Figure 3:**
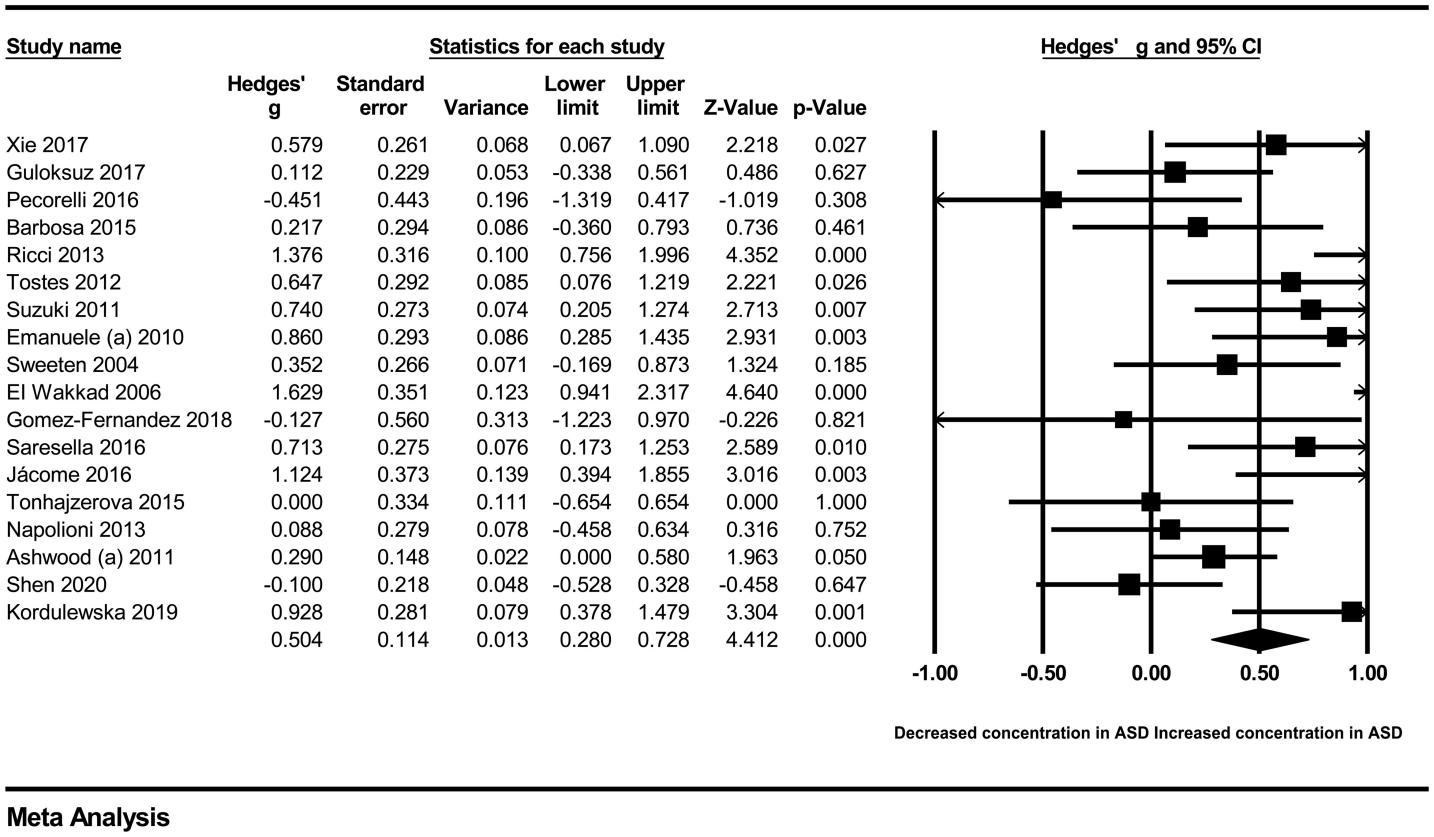
Forest plot for the random-effects meta-analysis (IL-1β).

**Figure 4:**
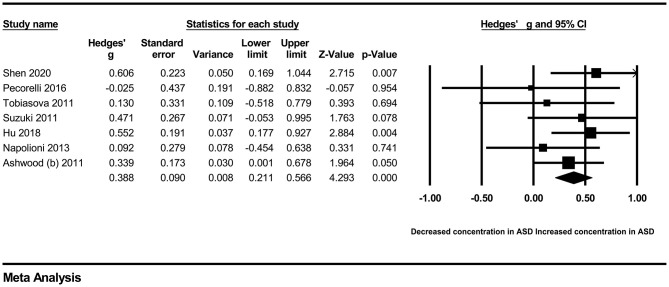
Forest plot for the fixed-effects meta-analysis (eotaxin-1).

**Figure 5:**
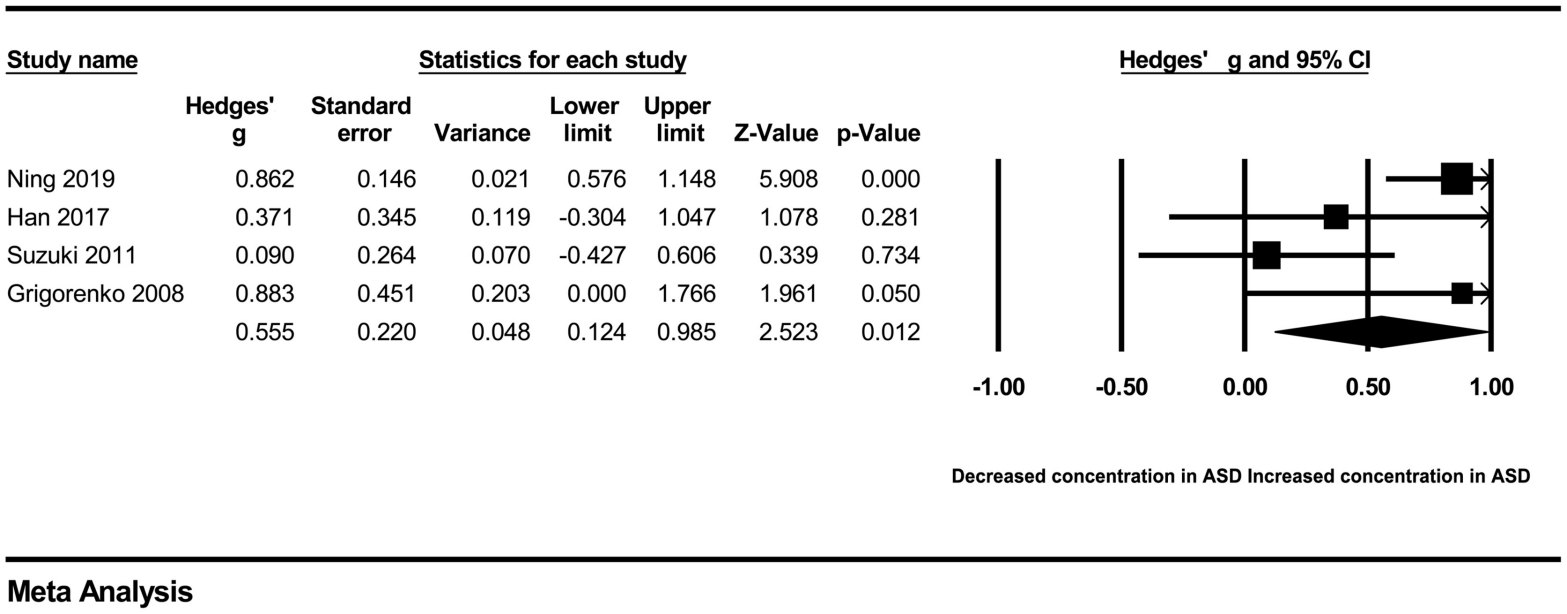
Forest plot for the random-effects meta-analysis (MIF).

**Figure 6:**
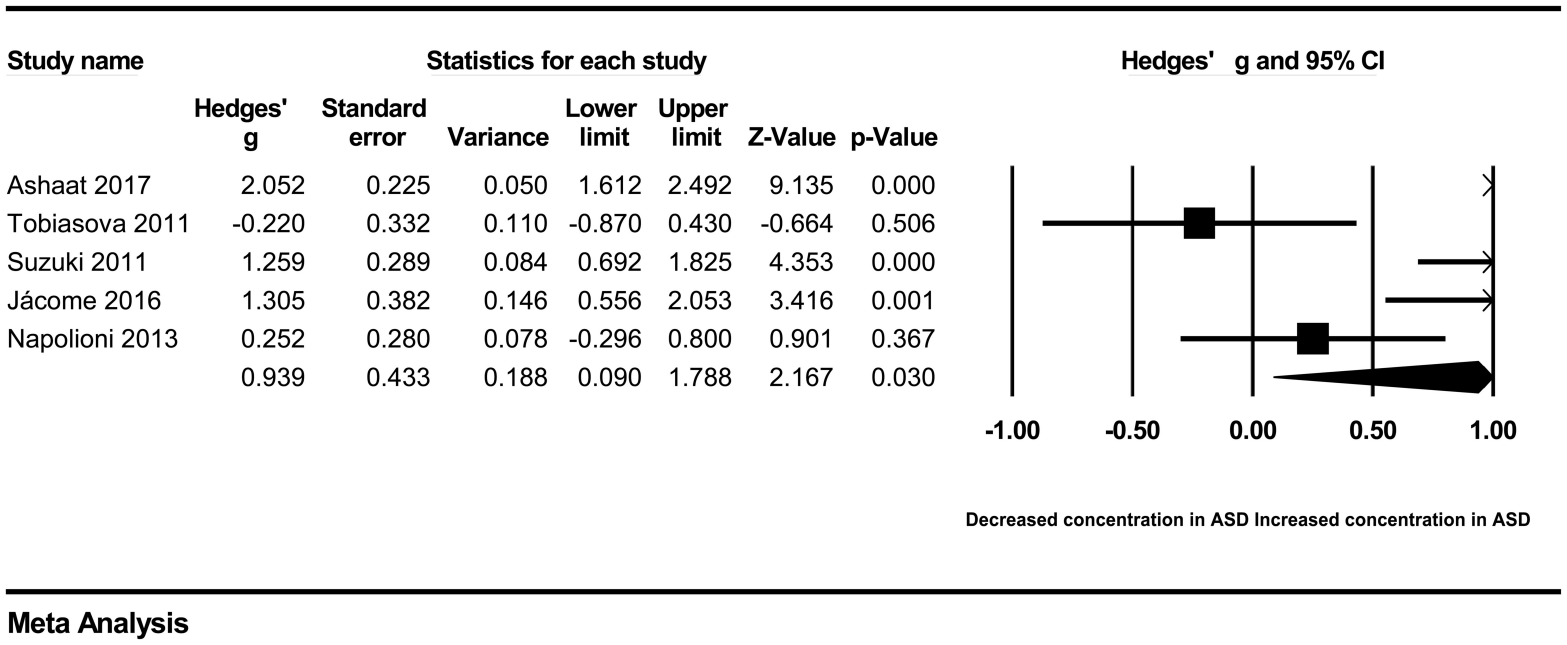
Forest plot for the random-effects meta-analysis (IL-12p70).

**Figure 7:**
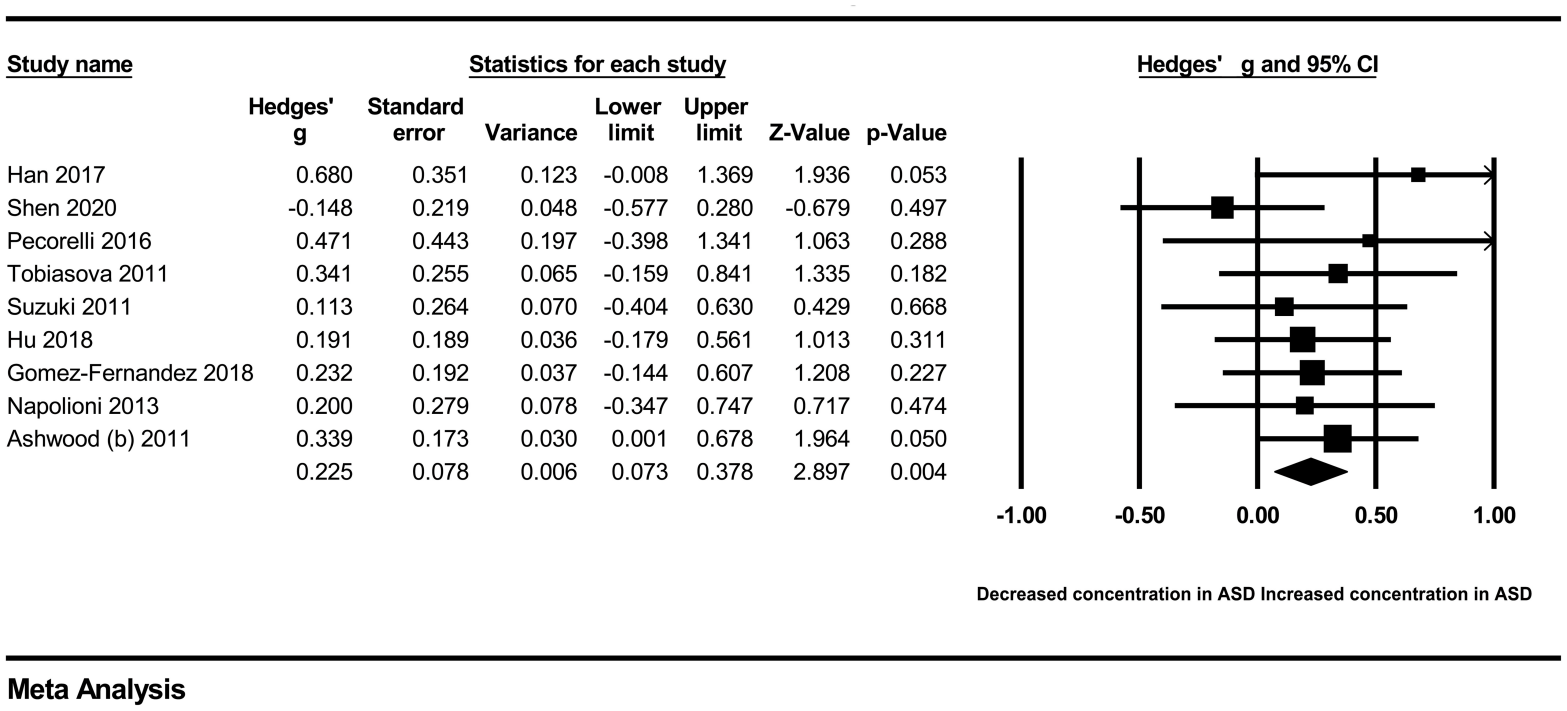
Forest plot for the fixed-effects meta-analysis (MCP1).

**Figure 8:**
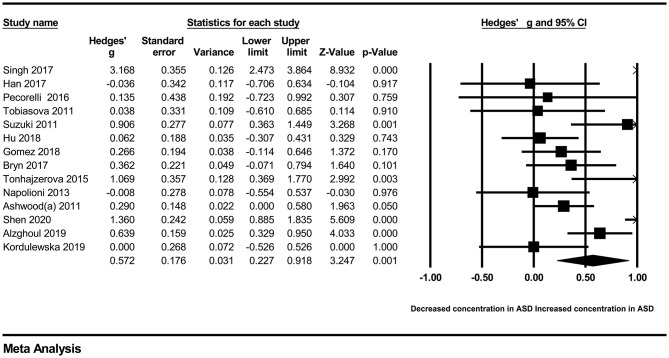
Forest plot for the random-effects meta-analysis (IL-8).

**Figure 9:**
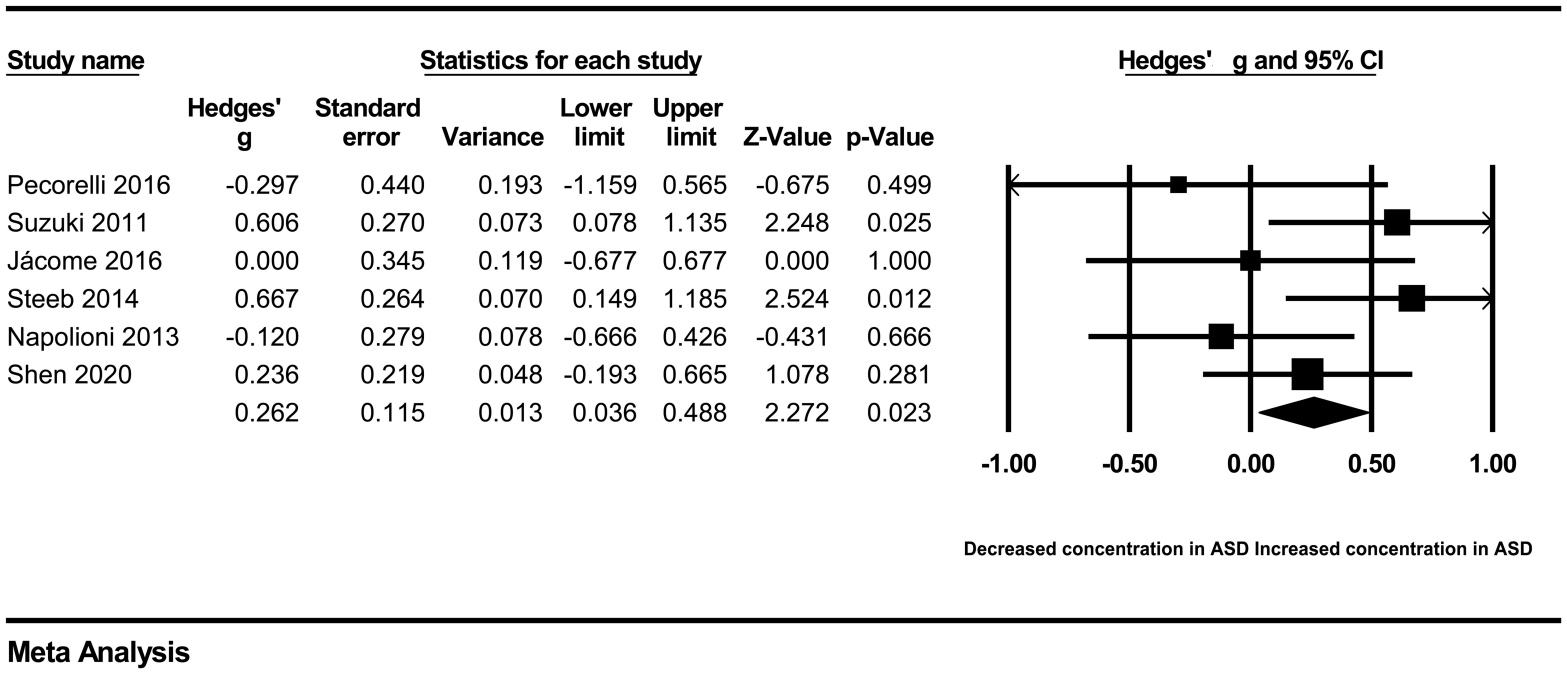
Forest plot for the fixed-effects meta-analysis (IL-7).

In our present meta-analysis, 289 studies were included to assess the association between the levels of 37 cytokines in peripheral blood and ASD. The results are shown in Supplementary Table 2. Eight cytokines were significantly elevated in the peripheral blood of ASD patients compared with controls (for IL-6: Hedges' *g* = 0.455, 95% CI 0.264–0.645, *P* < 0.001; for IL-1β: Hedges' *g* = 0.504, 95% CI 0.280–0.728, *P* < 0.001; for eotaxin-1: Hedges' *g* = 0.388, 95% CI 0.211–0.566, *P* < 0.001; for MIF: Hedges' *g* = 0.555, 95% CI 0.124–0.985, *P* = 0.012; for IL-12p70: Hedges' *g* = 0.939, 95% CI 0.090–1.788, *P* = 0.030; for MCP1: Hedges' *g* = 0.225, 95% CI 0.073–0.378, *P* = 0.004; for IL-8: Hedges' *g* = 0.572, 95% CI 0.227–0.918, *P* = 0.001; for IL-7: Hedges' *g* = 0.262, 95% CI 0.036–0.488, *P* = 0.023; Figures 2–9). In contrast, there were no obvious associations between the other 29 cytokine concentrations and ASD. Subgroup analyses were also performed in our meta-analysis based on different sample types. Six cytokines significantly increased in the plasma of ASD patients compared with controls (for IL-6: Hedges' *g* = 0.439, 95% CI 0.145–0.732, *P* = 0.003; for eotaxin-1: Hedges' *g* = 0.430, 95% CI 0.242–0.619, *P* < 0.001; for IL-8: Hedges' *g* = 0.542, 95% CI 0.235–0.849, *P* = 0.001; for MCP1: Hedges' *g* = 0.176, 95% CI 0.009–0.344, *P* = 0.039; for IL-1β: Hedges' *g* = 0.395, 95% CI 0.141–0.648, *P* = 0.002; for IL-12p70: Hedges' *g* = 1.224, 95% CI 0.409–2.038, *P* = 0.003), which was consistent with the overall analysis. Four cytokines were also significantly increased in the serum of ASD patients (for IL-6: Hedges' *g* = 0.511, 95% CI 0.301–0.722, *P* < 0.001; for IL-1β: Hedges' *g* = 0.726, 95% CI 0.345–1.106, *P* < 0.001; for IL-2: Hedges' *g* = 0.421, 95% CI 0.083–0.760, *P* = 0.015; for MCP1: Hedges' *g* = 0.461, 95% CI 0.094–0.827, *P* = 0.014). Moreover, patients with ASD had significantly higher IL-12 levels in plasma (Hedges' *g* = 1.763, 95% CI 0.431–3.095, *P* = 0.010) than controls.

### Heterogeneity Analysis

The I^2^ value and Q-test were used to evaluate heterogeneity across the studies. The results of eotaxin-1, MCP1, TNF-β, IL-2, IL-7, IL-9, and GM-CSF showed no obvious heterogeneity across studies. However, obvious heterogeneity was observed in the overall comparisons of the other 30 cytokines. Although subgroup analyses based on sample type were conducted, the heterogeneity did not decrease effectively (Supplementary Table 2). Due to the limited number of studies, heterogeneity analyses were mainly conducted for eight cytokines (IL-6, IFNγ, IL-8, TNF-α, IL-1β, IL-10, IL-17, and IL-4).

First, we tried to identify some potential moderators that may affect the heterogeneity of our meta-analysis. These potential moderators were usually divided into methodological and clinical sources. Due to the lack of information about methodological moderators, such as assay procedures, sample collection, separation and storage, and clinical moderators, such as gender (most data about these were inconsistent with the actual samples) and course of ASD, we could only perform meta-regression analyses on sample size, publication year, and age (only IL-17). However, meta-regression analyses suggested that the sample size, publication year, and age had no moderating effects on the results of our meta-analysis (Supplementary Figures 1–8). Then, we attempted to draw Galbraith plots, which revealed that some results deviated from the central for IL-6 (6 studies), IFNγ (3 studies), IL-8 (3 studies), TNF-α (8 studies), IL-1β (5 studies), IL-10 (3 studies), IL-17 (5 studies), and IL-4 (5 studies) (Supplementary Figures 9–16). After removing them separately, the heterogeneity across studies for each cytokine was significantly reduced. Associations between peripheral levels of these cytokines and ASD were not changed, except for TNF-α, IL-17, and IL-4. Patients with ASD had significantly elevated peripheral blood TNF-α and IL-4 levels compared with controls after removing the studies outside the boundaries in the Galbraith plot (for TNF-α: Hedges' *g* = 0.135, 95% CI 0.005–0.265, *P* = 0.042; for IL-4: Hedges' *g* = 0.213, 95% CI 0.023–0.402, *P* = 0.028). In addition, patients with ASD had significantly lower IL-17 levels in peripheral blood (Hedges' *g* = −0.208, 95% CI −0.398 to −0.017, *P* = 0.033) than controls (Table 2).

**Table 2:** Summary of meta-analysis results after removing studies outside the boundaries in Galbraith plot.

					**Tests of association**			**Tests of heterogeneity**			
**Cytokines**	**Studies (*n*)**	**Case (*n*)**	**Control (*n*)**	**Model**	**Hedges' g [95% CI]**	***Z***	***P*-value**	***Q*-value**	***P*-value**	***I^**2**^* (%)**	**Studies outside the boundaries in Galbraith plot**
IL-6	18	617	544	FE	0.409 [0.292–0.525]	6.866	0.000	21.467	0.206	20.808	(28, 44, 62, 73, 79, 81)
IFNγ	12	531	371	FE	0.054 [−0.080–0.189]	0.790	0.429	8.400	0.253	19.458	(24, 45, 52)
IL-8	11	531	407	FE	0.301 [0.170–0.432]	4.507	0.000	15.288	0.122	34.590	(60, 75, 82)
TNF-α	14	513	418	FE	0.135 [0.005–0.265]	2.032	0.042	18.403	0.143	29.360	(39, 61, 63, 72, 73, 76, 79, 82)
IL-1β	13	388	362	FE	0.360 [0.216–0.504]	4.905	0.000	16.042	0.190	25.113	(28, 50, 67, 80, 82)
IL-10	12	512	437	FE	−0.036 [−0.164–0.092]	−0.548	0.584	9.110	0.612	0.000	(41, 65, 67)
IL-17	7	276	185	FE	−0.208 [−0.398 to −0.017]	−2.130	0.033	10.317	0.122	41.845	(40, 42, 67, 71, 82)
IL-4	8	223	201	FE	0.213 [0.023–0.402]	2.200	0.028	9.596	0.213	27.052	(37, 80)

*FE, fixed-effects model*.

Sensitivity analyses indicated that the associations were generally similar when we removed any study for these cytokines, except for IL-4 (Supplementary Figure 17). The study (39) conducted by (37) could affect the statistically significant difference in the levels of IL-4 between patients with ASD and controls. This made sense, since this study also contributed to the heterogeneity.

### Publication Bias

Egger's test was carried out to quantitatively evaluate the publication bias in the included studies. The results did not show any clear evidence of obvious publication bias in these cytokines, except for IL-23 and IP-10. This is not surprising because only a few studies were included in the analysis of these cytokines. More studies should be carried out for further exploration. The results of our meta-analyses were unlikely to be affected by publication bias (Supplementary Table 2).

## Discussion

To the best of our knowledge, this study is the most comprehensive review and meta-analysis of the relationship between peripheral cytokines and ASD so far. Many cytokines were included in the meta-analysis for the first time. Overall, the results showed significant elevation of peripheral blood proinflammatory cytokine levels for IL-6, IL-1β, IL-7, and IL-12p70 in ASD patients compared with controls, which was in line with previous studies and our forecast. Persistent elevation of peripheral cytokines may reflect an ongoing inflammatory process in organisms. The presence of chronic inflammation in ASD has become increasingly well-documented. Large numbers of experimental studies have revealed that these cytokines play important roles in the development of ASD (83). Peripheral upregulated inflammation (IL-6, IL-1β, etc.) could worsen autistic symptoms in mice (84). These proinflammatory cytokines cross the blood-brain barrier and activate microglia. Dysfunctional microglia have a negative effect on synaptic pruning, influencing the signal transmission of neurons, which contributes to ASD development-a brain disorder characterized by an imbalance between inhibitory and excitatory responses (85). In addition, peripheral blood levels of MIF, eotaxin-1, MCP-1, and IL-8 were also markedly increased in ASD patients compared with controls. MIF, a conserved cytokine found as a homotrimer protein, directly or indirectly promotes the production or expression of a large panel of proinflammatory cytokines (such as IL-1β, IL-6, and IL-8) and mediates both acute and chronic inflammatory responses (9, 86, 87). Eotaxin-1, IL-8, and MCP-1 contribute to the selective recruitment of eosinophils, neutrophils, and leukocytes into inflammatory sites and participate in a skewed immune response (88, 89). Many previous studies have reported early neuroimmune dysfunction in children with ASD. These elevated chemokines indicate the existence of neuroimmune dysfunction, which is in line with the development of ASD (90). In fact, the exact mechanism of these altered cytokines on the pathogenesis of ASD is still unclear. Compared with the overall results, subgroup analyses suggested that the levels of IL-8 in serum and the levels of IL-7 in plasma of ASD patients were not significantly increased. This surprising finding may be caused by different components in serum and plasma. The sensitivity of the same chemokine may vary in different blood components. Similarly, IL-2 was heightened in the serum of ASD patients compared with the controls. In addition, we observed that IL-12 was greatly elevated in the plasma of ASD patients. However, we should note that only a few studies were included in the analysis of each cytokine. More clinical studies are needed to further demonstrate these results.

Generally, these cytokines in peripheral blood may be helpful for the diagnosis and treatment of ASD. Our study strengthened the clinical evidence of an increased inflammatory response in ASD patients. There was no clear evidence of significant alteration of peripheral blood levels of the remaining 27 cytokines in ASD patients compared with controls. Since there were fewer than three studies on each of the other 39 cytokines, we only provided some basic information about these studies. More clinical studies with larger sample sizes are required for analysis.

There was obvious heterogeneity in our analysis for most cytokines, which may be caused by different processing methods and clinical samples. We attempted to find the cause of heterogeneity across studies by subgroup analysis and meta-regression analyses. However, limited information, such as sample size, publication year, and age, could not effectively explain the generation of heterogeneity. Galbraith plots helped us distinguish studies contributing to the heterogeneity in our meta-analysis. After excluding these studies, heterogeneity decreased effectively. Moreover, patients with ASD had significantly increased peripheral blood levels of TNF-α, IL-17, and IL-4 compared with controls. Excessive TNF-α has been proven to be involved in autistic behaviors, such as impaired social interaction and repetitive behaviors (91), which may also participate in the imbalance between the inhibitory and excitatory behaviors of the brain. This was consistent with our result. However, we noted that the results of peripheral IL-17 and IL-4 were contrary to other altered proinflammatory and anti-inflammatory cytokines in our meta-analysis. These results suggested that peripheral altered IL-17 and IL-4 may be the result of negative feedback conditions. Therefore, TNF-α, IL-17, and IL-4 may also be members of the abnormal cytokine profile in peripheral blood of ASD patients, and it was hidden by heterogeneity.

ASD is a group of complex, pervasive neurodevelopmental disorders with unknown etiology. In the ASD brain, an exaggerated inflammatory response may be a hallmark event to explain the pathologic conditions of ASD (92, 93). In our study, 11 cytokines were considered to be involved in abnormal cytokine profiles in the peripheral blood of patients with ASD. However, we did not know the specific relationship of these cytokines in peripheral blood and brain. It was difficult to discern causality. Some studies have suggested that peripheral inflammatory cytokines may enter the central nervous system, leading to severe inflammation when the blood-brain barrier is weak (94). In addition, how many cytokines in the brain can be represented by the levels of cytokines in peripheral blood is questionable, although it is generally believed that cytokines in peripheral blood are associated with cytokines in the central nervous system. Therefore, further basic and clinical studies are needed to solve the above two problems.

With this research, IL-6, IL-1β, IL-12p70, MIF, eotaxin-1, MCP-1, IL-8, IL-7, IL-2, IL-12, TNF-α, IL-17, and IL-4 may be identified as a series of potential biomarkers for ASD in peripheral blood. This may provide some important clues for ASD therapeutic discovery. Of course, there were some limitations that should be pointed out. First, although many cytokines were included in our meta-analysis, the sample size was moderate, and the problem of heterogeneity remained unresolved due to the lack of information in many included studies. For some cytokines, we could only make a systemic summary. Second, further research is necessary to evaluate the relationship between peripheral cytokines and cytokines in the brain, which could help us understand the pathogenesis of ASD. Third, although we analyzed the correlation between some peripheral cytokines and ASD, the specific mechanism of most altered cytokines in ASD is still unclear. More studies are urgently needed to provide an exact explanation.

## Conclusions

In our meta-analysis, we found that the levels of peripheral IL-6, IL-1β, IL-12p70, MIF, eotaxin-1, MCP-1, IL-8, IL-7, IL-2, IL-12, TNF-α, IL-17, and IL-4 were significantly changed in ASD patients compared with controls. These findings strengthened the clinical evidence of ASD with an abnormal inflammatory response. These cytokines may be a series of potential biomarkers for ASD in peripheral blood.

## Data Availability Statement

The original contributions presented in the study are included in the article/Supplementary Material, further inquiries can be directed to the corresponding author/s.

## Author Contributions

JG conceived the study. JG, HZhao, and HZhang collected the data and drafted the manuscript. SL, WL, and YJ revised the manuscript. All authors contributed to the article and approved the submitted version.

## Conflict of Interest

The authors declare that the research was conducted in the absence of any commercial or financial relationships that could be construed as a potential conflict of interest.

## Abbreviations

ASD: autism spectrum disorder
MIF: Macrophage migration inhibitory factor
MCP-1: Monocyte chemotactic protein-1
TNF-α: Tumor necrosis factor-α
SD: standard deviation
ESs: effect sizes
SMDs: standardized mean differences
CIs: confidence intervals.
