# Supplementary material for: Association of Peripheral Blood Levels of Cytokines With Autism Spectrum Disorder: A Meta-Analysis
Source: Front Psychiatry. 2021 Jul 2;12:670200. doi: 10.3389/fpsyt.2021.670200 (PMC8283413; doi:10.3389/fpsyt.2021.670200)
Supplement: Supplementary file 1 [file Table_1.docx]

**Supplementary Table 1. Characteristics of the included studies in the meta-analysis.**

| **First author** | **Country** | **Ethnicity** | **Comparition** | **Diagnostic criteria** | **Sample type** | **Sources** | | **Detection method** | **Largest sample size** | | **Data type** |
| --- | --- | --- | --- | --- | --- | --- | --- | --- | --- | --- | --- |
| **Publication year** |  |  |  |  |  | **Case** | **Control** |  | **Case** | **Control** |  |
| Shen 2020 | China | Chinese | ASD/TD children | DSM-IV-TR | Plasma | Special education schools and hospital outpatient services in Changsha, Hunan Province | Regular kindergarten classes | Invitrogen’s Human Cytokine Twenty-Five-Plex Antibody Bead Kit | 45 | 38 | Mean±SD |
| Kordulewska 2019 | Poland | Not described | ASD/TD children | DSM-5 | Serum | The Center for Diagnosis, Treatment and Therapy of Autism at the Regional Children's Hospital in Olsztyn, Poland | The Center for Diagnosis, Treatment and Therapy of Autism at the Regional Children's Hospital in Olsztyn, Poland | ELISA | 42 | 20 | Sample size and P-value |
| Alzghoul 2019 | Jordan | Not described | ASD/TD children | DSM-5 | Plasma | Pediatric clinics and local special healthcare centers | Visitors to our  local university hospital | ELISA | 80 | 86 | Mean±SD  Sample size and P-value |
| Ning 2019 | China | Chinese | ASD/TD children | DSM-5 | Serum | Patients from the Affiliated Hospital of Jining Medical University | A kindergarten near the Affiliated Hospital of Jining Medical University, Jining, China | ELISA | 102 | 102 | Mean±SD |
| Hu 2018 | China | Chinese | ASD/TD children | DSM-5 | Plasma | Patients from the Children’s Hospital of Fudan University | A local kindergarten | MILLIPLEX MAP | 87 | 41 | Sample size and P-value |
| Gomez-Fernandez 2018 | Spain | Not described | ASD/Children diagnosed with phimosis and hernias, lacking any other pathology | DSM-5 | Plasma | Patients from the Department of Child and Adolescent Clinical Psychiatry and Psychology of Reina Sofia University Hospital | Reina Sofia University Hospital | LINCOplex assay kits and Luminex xMAP Detection Technology | 54 | 54 | Sample size and P-value |
| Xie 2017 | China | Chineses | ASD/TD children | DSM-IV-TR | Serum | The three training schools for autistic children in Chengdu, China | A primary school in Chengdu, China | MILLIPLEX MAP | 32 | 28 | Mean±SD |
| Singh 2017 | USA | Not described | ASD/TD children | DSM-IV | Serum | The Johnson Center clinic, or through the use of informational study flyers circulated around Austin, TX | The Johnson Center clinic, or through the use of informational study flyers circulated around Austin, TX | The MSD platform | 36 | 35 | Mean±SD |
| Han 2017 | China | Not described | ASD/TD children | DSM-5 | Serum | Patients in the database at the Neuropsychology Laboratory of the Chinese University of Hong Kong | Not described | ELISA | 22 | 13 | Mean±SD |
| Guloksuz 2017 | USA | Not described | ASD/TD children | DSM-IV | Plasma | Long-term follow-up outpatients of the Autism Clinic of the Child and Adolescent Psychiatry Department at Istanbul University School of Medicine, Istanbul, Turkey | The Well Child Outpatient Clinic | MILLIPLEX MAP and ELISA | 40 | 35 | Mean±SD |
| Bryn 2016 | Norway | Not described | ASD/TD children | the Autism Diagnostic Interview–Revised, the Autism Diagnostic Observation Schedule, and the ICD-10 | Serum | An epidemiological survey covering two counties in Norway, Oppland and Hedmark | The Department of Pediatrics of the Innlandet Hospital Trust at Lillehammer | Bio-Plex Xmap technology | 65 | 30 | Sample size and P-value |
| Ashaat 2017 | Egypt | Not described | ASD/healthy children | ICD-10 | Plasma | The Clinical Genetics Clinic, National Research Centre, Egypt | Not described | ELISA | 60 | 60 | Mean±SD |
| Pecorelli 2016 | Italy | Not described | ASD/healthy control | DSM-5 | Serum | the Rett Syndrome National Reference Centre of the University Hospital of Siena (Azienda Ospedaliera Universitaria Senese) | Not described | Multiplex kit and ELISA | 12 | 8 | Mean±SD |
| Ghaffari 2016 | Iran | Iranian | ASD/healthy children | DSM-IV | Serum | The state of Khuzestan | The state of Khuzestan | ELISA | 30 | 30 | Mean±SD |
| El-Ansary 2016 | Saudi Arabia | Not described | ASD/healthy children | ADI-R/ADOS/3DI | Plasma | The ART Center (Autism Research & Treatment Center) clinic | The pediatric clinic at King Saud medical city | ELISA | 35 | 38 | Mean±SD |
| Saresella 2016 | Italy | Not described | ASD/healthy children | DSM-IV | Serum | Not described | Not described | ELISA | 25 | 30 | Sample size and P-value |
| Jácome 2016 | Cuba | Not described | ASD/healthy children | DSM-IV-TR | Plasma | Not described | Not described | ELISA | 17 | 15 | Sample size and P-value |
| Businaro 2016 | Italy | Not described | ASD/healthy controls | DSM-IV-TR | Serum | The Center for Autism of Hospital of Chiaromonte/Lagonegro (Potenza) and at the Pediatrics Neuropsychiatry Department of Matera | Not described | ELISA | 29 | 29 | Mean±SD |
| Shaker 2016 | Egypt | Not described | ASD/healthy children | ICD-10 | Serum | The child psychiatry outpatient clinics of the Institute of Psychiatry and the Institute of childhood studies, Ain Shams University, Cairo, Egyp | The child psychiatry outpatient clinics of the Institute of Psychiatry and the Institute of childhood studies, Ain Shams University, Cairo, Egyp | ELISA | 30 | 22 | Sample size and P-value |
| Yang 2015 | China | Chinese | ASD/healthy children | DSM-IV-TR | Plasma | Area schools or autism outreach groups in Shanghai, China | Area schools or autism outreach groups in Shanghai, China | ELISA | 35 | 32 | Sample size and P-value |
| Yan 2015 | China | Chinese | ASD/children | DSM-IV | Plasma | Not described | The outpatient medical center | A standard laboratory method | 75 | 75 | Mean±SD |
| Barbosa 2015 | Brazil | Not described | ASD/healthy children | DSM-IV-TR | Plasma | The Child Psychiatry Clinic from the University Hospital, Universidade Federal de Minas Gerais, Brazil | The local community | ELISA | 30 | 18 | Mean±SD |
| Tonhajzerova 2015 | Slovakia | Not described | ASD/healthy children | DSM-5 | Plasma | Not described | Primary school | ELISA | 15 | 20 | Sample size and P-value |
| Mostafa 2015 | Egypt | Not described | ASD/healthy children | DSM-IV | Serum | The Pediatric Neuropsychiatric Clinic, Faculty of Medicine of Ain Shams University, Cairo, Egypt | The Outpatients Clinic, Children’s Hospital, Faculty of Medicine, Ain Shams University | The quantitative sandwich enzyme immunoassay technique | 62 | 62 | Sample size and P-value |
| Tsilioni 2015 | USA | Caucasian | ASD/healthy children | DSM-IV-TR and ADOS | Serum | The Attikon General Hospital, Athens Medical School, Athens, Greece | Not described | ELISA | 40 | 13 | Mean±SD/Sample size and P-value |
| EI Gohary 2015 | Egypt | Not described | ASD/healthy children | DSM-IV-TR | Plasma | Outpatient clinic of Pediatric Neuropsychiatry and Phoniatrics of Tanta University Hospital | Not described | ELISA | 30 | 30 | Mean±SD |
| Ibrahim 2015 | Egypt | Not described | ASD/healthy children | DSM-IV-TR | Serum | The Child and Adolescent Outpatients Clinic at Al Hadra University Hospital | The Child and Adolescent Outpatients Clinic at Al Hadra University Hospital | ELISA | 20 | 20 | Mean±SD |
| El-Ansary 2014 | Saudi Arabia | Not described | ASD/healthy children | ADI-R/ADOS/3DI | Plasma | The ART Center (Autism Research & Treatment Center) clinic | Well-baby Clinic at King Khaled University hospital | ELISA | 20 | 19 | Mean±SD |
| Steeb 2014 | UK | Not described | Asperger syndrome/healthy control | DSM-IV-TR | Serum | The Cambridge Autism Research Centre database of volunteers, the National Autistic Society (UK) and local autism social groups in the UK | General population | Multiplex immunoassay analysis | 30 | 29 | Sample size and P-value |
| Tsilioni 2014 | USA | Caucasian | ASD/healthy children | DSM-IV-TR | Serum | The Attikon General Hospital, Athens Medical School, Athens, Greece | Not described | ELISA | 38 | 13 | Mean±SD |
| Russo 2014 | USA | Not described | ASD/healthy children | DSM-IV | Plasma | The Health Research Institute (HRI) | The Health Research Institute (HRI) | ELISA | 40 | 39 | Mean±SD |
| Russo 2013 | USA | Not described | ASD/neurotypical children | DSM-IV | Plasma | The Health Research Institute/Pfeiffer Treatment Center | The Health Research Institute/Pfeiffer Treatment Center | ELISA | 38 | 40 | Mean±SD |
| Ricci 2013 | Italy | Not described | ASD/healthy children | DSM-IV-TR | Serum | The Center for Autism of hospital of Chiaromonte/Lagonegro (PZ) and at the Pediatrics Neuropsychiatry Department of Matera | Not described | ELISA | 29 | 29 | Mean±SD |
| Napolioni 2013 | USA | Caucasian, Hispanic, Caucasian-Hispanic, Asian | ASD/healthy children | DSM-IV | Plasma | The Southwest Autism Research and Resource Center (SAR RC) (Phoenix , AZ , USA) | Not described | ELISA | 25 | 25 | Sample size and P-value |
| Hashim 2013 | Egypt | Egyptian | ASD/TD children | DSM-IV | Plasma | The Pediatric and Psychiatric Outpatients Clinics, Faculty of Medicine, Zagazig University, Zagazig, Egypt | The Pediatric and Psychiatric Outpatients Clinics | ELISA | 50 | 50 | Sample size and P-value |
| Ramsey 2013 | UK | Not described | ASD/unaffected siblings | APA and DSM-IV-TR | Serum | Karakter Child and Adolescent Psychiatry and the Radboud University Nijmegen Medical Center in Nijmegen, The Netherlands | Siblings recruited from the same families | Multiplex immunoassay analysis | 37 | 37 | Sample size and P-value |
| AL-Ayadhi 2013 | Saudi Arabia | Not described | ASD/healthy children | DSM-IV | Serum | The Autism Research and Treatment Center, Faculty of Medicine, King Saud University, Riyadh, Saudi Arabia | The healthy older siblings of the healthy infants who attend the Well Baby Clinic, King Khalid University Hospital, Faculty of Medicine, King Saud University, Riyadh, Saudi Arabia | The quantitative sandwich enzyme immunoassay technique | 56 | 32 | Sample size and P-value |
| AL-Ayadhi 2012 | Saudi Arabia | Not described | ASD/healthy children | DSM-IV | Serum | The Autism Research and Treatment Center, King Khalid University Hospital (Riyadh, Saudi Arabia) | The healthy older siblings of healthy infants who were attending the Well Baby Clinic at King Khalid University Hospital | ELISA | 45 | 40 | Sample size and P-value |
| El-Ansary 2012 | Saudi Arabia | Not described | ASD/healthy children | ADI-R/ADOS/3DI | Plasma | The ART Center (Autism Research & Treatment Center) clinic | Well-baby Clinic at King Khaled University hospital | ELISA | 20 | 19 | Mean±SD |
| Tostes 2012 | Brazil | Not described | ASD/healthy children | DSM-IV | Plasma | Not described | A public school | ELISA and flow cytometry using Cell Quest software | 24 | 24 | Mean±SD |
| Manzardo 2012 | USA | Caucasian | ASD/healthy unrelated siblings | ADOS and/or ADIR | Plasma | The Autism Genetic Research Exchange | The Autism Genetic Research Exchange | Multiplex sandwich immunoassays | 99 | 40 | Sample size and P-value |
| Tobiasova 2011 | USA | Not described | ASD/healthy children | DSM-IV | Serum | The Research Units on Pediatric Psychopharmacology Autism Network | The New Haven, Connecticut area | Millipore Luminex based kit | 21 | 15 | Mean±SD |
| Suzuki 2011 | Japan | Japanese | ASD/healthy children | DSM-IV | Plasma | Born and living in restricted areas of central Japan, including Aichi, Gifu, and Shizuoka prefectures | Born and living in restricted areas of central Japan, including Aichi, Gifu, and Shizuoka prefectures | Multiplex kits | 28 | 28 | Mean±SD |
| El-Ansary 2011 | Saudi Arabia | Not described | ASD/healthy children | ADI-R/ADOS/3DI | Plasma | The ART Center (Autism Research & Treatment Center) clinic | Well baby Clinic at King Khaled University hospital | Not described | 25 | 16 | Mean±SD |
| Ashwood (b) 2011 | USA | Not described | ASD/TD children | ADI-R and ADOS | Plasma | The population-based case-control CHARGE (Childhood Autism Risk from Genetics and Environment) study | The population-based case-control CHARGE (Childhood Autism Risk from Genetics and Environment) study | Human bead immunoassays using Luminex technology | 80 | 58 | Sample size and P-value |
| Ashwood (a) 2011 | USA | Not described | ASD/TD children | DSM-IV | Plasma | The population-based case-control CHARGE (Childhood Autism Risk from Genetics and Environment) study | The population-based case-control CHARGE (Childhood Autism Risk from Genetics and Environment) study | Human multiplexing bead immunoassays | 97 | 87 | Sample size and P-value |
| Kajizuka 2010 | Japan | Japanese | ASD/healthy children | ADI-R, DSM-IV-TR, ICD-10 and DSM-IV | Serum | Advocacy groups in cooperation with the Asperger Society of Japan in Nagoya and Hamamatsu | Hamamatsu City | ELISA | 31 | 31 | Mean±SD |
| Emanuele 2010 | Italy | Caucasian Italian | Severe autism/healthy adult | ADI-R | Serum | Cascina Rossago (Ponte Nizza, Italy), a residential farm community for adult individuals with autism | Laboratory personnel and community volunteers | ELISA | 22 | 28 | Mean±SD |
| Russo 2009 | USA | Not described | ASD/non autistic controls | Not described | Serum | The Thoughtful House, Austin, Texas | The Autism Genetic Resource Exchange—AGRE | ELISA | 40 | 20 | Mean±SD |
| Enstrom 2008 | USA | Not described | ASD/TD children | ADI-R and ADOS | Plasma | The CHARGE (Childhood Autism Risk from Genetics and Environment) study conducted at the UC Davis M.I.N.D. Institute | Not described | ELISA | 40 | 20 | Sample size and P-value |
| Ashwood 2008 | USA | Not described | ASD/general population | ADI-R, ADOS and DSM-IV | Plasma | The CHARGE (Childhood Autism Risk from Genetics and Environment) study conducted at the UC Davis M.I.N.D. Institute | Not described | ELISA | 75 | 36 | Sample size and P-value |
| Grigorenko 2008 | USA | Dutch | ASD/unaffected siblings | ADI-R and ADOS | Plasma | The Accare Center | Not described | ELISA | 10 | 10 | Mean±SD |
| Sugihara 2007 | Japan | Japanese | High-functioning autism/subjects who had no developmental delay and no history of psychiatric disorders or treatment | ADI-R and DSM-IV | Serum | Not described | Not described | ELISA | 17 | 18 | Mean±SD |
| Okada 2007 | Japan | Japanese | Autism/healthy adult | ADI-R and DSM-IV | Serum | Advocacy groups in Nagoya and Hamamatsu cities | Hamamatsu City | ELISA | 19 | 21 | Mean±SD |
| EI Wakkad 2006 | Egypt | Not described | ASD/healthy children | DSM-IV | Plasma | Not described | Not described | Enzyme Amplified Sensitivity Immunoassay | 21 | 21 | Mean±SD |
| Al-Ayadhi 2005 | Saudi Arabia | Not described | ASD/healthy children | E-2 | Serum | Riyadh area in Saudi Arabia | Riyadh area in Saudi Arabia | ELISA | 77 | 77 | Sample size and P-value |
| Sweeten 2004 | USA | Not described | ASD/healthy children | ADI-R and DSM-IV | Plasma | The Christian Sarkine Autism Treatment Center at the James Whitcomb Riley Hospital for Children in Indianapolis, Indiana | The surrounding community via newsletters and flyers | ELISA | 29 | 27 | Mean±SD |
| Croonenberghs 2002 | Belgium | Caucasian | ASD/healthy children | DSM-IV | Serum | Not described | Not described | ELISA | 13 | 13 | Mean±SD |
| Denney 1996 | USA | White | ASD/healthy children | DSM-III-R | Plasma | The Kansas City/Topeka area in USA | The Kansas City/Topeka area in USA | ELISA | 10 | 10 | Mean±SD |
| Singh 1996 | USA | Not described | ASD/normal children | DSM-IIIR | Plasma | Ann Arbor, USA | Ann Arbor, USA | ELISA | 20 | 20 | Sample size and P-value |
| Singh 1991 | USA | Caucasian | ASD/healthy children | DSM-IIIR | Serum | Not described | The same reginal population as the autistic children | ELISA | 23 | 33 | Mean±SD |
|  |  |  |  |  |  |  |  |  |  |  |  |
