# Supplementary material for: Association of Peripheral Blood Levels of Cytokines With Autism Spectrum Disorder: A Meta-Analysis
Source: Front Psychiatry. 2021 Jul 2;12:670200. doi: 10.3389/fpsyt.2021.670200 (PMC8283413; doi:10.3389/fpsyt.2021.670200)
Supplement: Supplementary file 2 [file Table_2.docx]

**Supplementary Table 2.** **Summary of meta-analysis results.**

|  |  |  |  |  | **Tests of association** | | | | **Tests of heterogeneity** | | | **Publication bias** |
| --- | --- | --- | --- | --- | --- | --- | --- | --- | --- | --- | --- | --- |
| **Cytokines** | **Groups** | **Studies (n)** | **Case (n)** | **Control (n)** | **Model** | **Hedges’ g [95%CI]** | **Z** | **P-value** | **Q-value** | **P-value** | **I^2^ (%)** | **Egger’s P-value** |
| **MIF** | All | 4 | 162 | 153 | RE | 0.555 [0.124-0.985] | 2.523 | 0.012 | 7.543 | 0.056 | 60.228 | 0.507 |
| **IL-6** | All | 24 | 1034 | 903 | RE | 0.455 [0.264-0.645] | 4.674 | <0.001 | 90.858 | <0.001 | 74.686 | 0.841 |
|  | Plasma | 13 | 630 | 560 | RE | 0.439 [0.145-0.732] | 2.926 | 0.003 | 71.109 | <0.001 | 83.125 |  |
|  | Serum | 11 | 404 | 343 | RE | 0.511 [0.301-0.722] | 4.757 | <0.001 | 17.446 | 0.065 | 42.682 |  |
| **Eotaxin-1** | All | 7 | 298 | 213 | FE | 0.388 [0.211-0.566] | 4.293 | <0.001 | 4.495 | 0.610 | 0.000 | 0.144 |
|  | Plasma | 5 | 265 | 190 | FE | 0.430 [0.242-0.619] | 4.468 | <0.001 | 2.801 | 0.592 | 0.000 |  |
| **IFNγ** | All | 15 | 595 | 434 | RE | 0.275 [-0.055-0.605] | 1.634 | 0.102 | 86.495 | <0.001 | 83.814 | 0.070 |
|  | Plasma | 11 | 432 | 359 | RE | 0.397 [-0.041-0.836] | 1.775 | 0.076 | 85.154 | <0.001 | 88.257 |  |
|  | Serum | 4 | 163 | 75 | FE | 0.034 [-0.244-0.312] | 0.240 | 0.810 | 0.768 | 0.857 | 0.000 |  |
| **IL-1RA** | All | 8 | 352 | 202 | RE | 0.191 [-0.217-0.598] | 0.917 | 0.359 | 34.042 | <0.001 | 79.437 | 0.671 |
|  | Plasma | 4 | 185 | 132 | RE | 0.491 [-0.114-1.096] | 1.592 | 0.111 | 19.713 | <0.001 | 84.782 |  |
|  | Serum | 4 | 167 | 70 | FE | -0.220 [-0.503-0.063] | -1.523 | 0.128 | 3.237 | 0.357 | 7.313 |  |
| **IL-1α** | All | 4 | 229 | 112 | RE | -0.014 [-0.365-0.336] | -0.081 | 0.935 | 6.458 | 0.091 | 53.548 | 0.271 |
|  | Plasma | 3 | 152 | 93 | FE | -0.163 [-0.425-0.100] | -1.211 | 0.226 | 3.595 | 0.166 | 44.365 |  |
| **IL-8** | All | 14 | 627 | 500 | RE | 0.572 [0.227-0.918] | 3.247 | 0.001 | 95.757 | <0.001 | 86.424 | 0.324 |
|  | Plasma | 8 | 429 | 379 | RE | 0.542 [0.235-0.849] | 3.455 | 0.001 | 30.135 | <0.001 | 76.771 |  |
|  | Serum | 6 | 198 | 121 | RE | 0.606 [-0.296-1.508] | 1.316 | 0.188 | 65.559 | <0.001 | 92.373 |  |
| **MCP1** | All | 9 | 430 | 284 | FE | 0.225 [0.073-0.378] | 2.897 | 0.004 | 5.769 | 0.673 | 0.000 | 0.406 |
|  | Plasma | 6 | 319 | 244 | FE | 0.176 [0.009-0.344] | 2.062 | 0.039 | 3.249 | 0.662 | 0.000 |  |
|  | Serum | 3 | 111 | 40 | FE | 0.461 [0.094-0.827] | 2.460 | 0.014 | 0.611 | 0.737 | 0.000 |  |
| **MIP-1α** | All | 8 | 397 | 253 | RE | -0.041 [-0.460-0.379] | -0.190 | 0.850 | 44.092 | <0.001 | 84.124 | 0.562 |
|  | Plasma | 6 | 364 | 230 | RE | -0.027 [-0.525-0.471] | -0.106 | 0.915 | 41.921 | <0.001 | 88.073 |  |
| **MIP-1β** | All | 8 | 397 | 253 | RE | 0.135 [-0.307-0.577] | 0.598 | 0.550 | 48.798 | <0.001 | 85.655 | 0.347 |
|  | Plasma | 6 | 364 | 230 | RE | 0.181 [-0.357-0.720] | 0.660 | 0.509 | 48.443 | <0.001 | 89.678 |  |
| **TGF-β** | All | 7 | 303 | 210 | RE | -0.075 [-0.858-0.708] | -0.187 | 0.852 | 96.699 | <0.001 | 93.795 | 0.504 |
|  | Plasma | 5 | 262 | 176 | RE | 0.233 [-0.694-1.160] | 0.493 | 0.622 | 77.458 | <0.001 | 94.836 |  |
| **TNF-α** | All | 22 | 873 | 698 | RE | 0.114 [-0.141-0.369] | 0.875 | 0.381 | 124.219 | <0.001 | 83.094 | 0.180 |
|  | Plasma | 14 | 601 | 528 | RE | 0.045 [-0308-0.398] | 0.252 | 0.801 | 106.769 | <0.001 | 87.824 |  |
|  | Serum | 8 | 272 | 170 | RE | 0.230 [-0.083-0.544] | 1.439 | 0.150 | 17.377 | 0.015 | 59.716 |  |
| **TNF-β** | All | 3 | 90 | 90 | FE | 0.166 [-0.124-0.456] | 1.120 | 0.263 | 3.386 | 0.184 | 40.931 | 0.247 |
| **VEGF** | All | 5 | 179 | 123 | RE | -0.015 [-0.416-0.386] | -0.073 | 0.942 | 10.611 | 0.031 | 62.304 | 0.872 |
|  | Serum | 3 | 64 | 54 | FE | -0.195 [-0.553-0.164] | -1.063 | 0.288 | 2.121 | 0.346 | 5.690 |  |
| **EGF** | All | 3 | 212 | 99 | RE | -0.601 [-1.687-0.486] | -1.084 | 0.278 | 33.540 | <0.001 | 94.037 | 0.895 |
| **IL-1β** | All | 18 | 534 | 484 | RE | 0.504 [0.280-0.728] | 4.412 | <0.001 | 49.874 | <0.001 | 65.914 | 0.330 |
|  | Plasma | 12 | 380 | 342 | RE | 0.395 [0.141-0.648] | 3.047 | 0.002 | 29.060 | 0.002 | 62.147 |  |
|  | Serum | 6 | 154 | 142 | RE | 0.726 [0.345-1.106] | 3.739 | <0.001 | 12.261 | 0.031 | 59.221 |  |
| **HGF** | All | 5 | 160 | 135 | RE | -0.308 [-1.079-0.463] | -0.782 | 0.434 | 39.029 | <0.001 | 89.751 | 0.639 |
|  | Serum | 3 | 78 | 53 | RE | -0.725 [-1.935-0.484] | -1.176 | 0.240 | 20.565 | <0.001 | 90.275 |  |
| **IL-10** | All | 15 | 585 | 505 | RE | -0.159 [-0.484-0.166] | -0.959 | 0.337 | 93.230 | <0.001 | 84.983 | 0.425 |
|  | Plasma | 9 | 391 | 376 | RE | -0.263 [-0.751-0.225] | -1.055 | 0.291 | 84.112 | <0.001 | 90.489 |  |
|  | Serum | 6 | 194 | 129 | FE | -0.029 [-0.254-0.195] | -0.256 | 0.798 | 8.708 | 0.121 | 12.580 |  |
| **IL-17** | All | 12 | 471 | 366 | RE | 0.158 [-0.217-0.533] | 0.825 | 0.410 | 74.338 | <0.001 | 85.203 | 0.545 |
|  | Plasma | 8 | 305 | 271 | RE | 0.164 [-0.330-0.658] | 0.650 | 0.515 | 58.539 | <0.001 | 88.042 |  |
|  | Serum | 4 | 166 | 95 | RE | 0.164 [-0.427-0.756] | 0.545 | 0.586 | 13.977 | 0.003 | 78.536 |  |
| **IL-2** | All | 7 | 258 | 243 | FE | 0.094 [-0.081-0.269] | 1.056 | 0.291 | 7.467 | 0.280 | 19.652 | 0.573 |
|  | Plasma | 4 | 191 | 174 | FE | -0.025 [-0.228-0.179] | -0.236 | 0.813 | 0.772 | 0.856 | 0.000 |  |
|  | Serum | 3 | 67 | 69 | FE | 0.421 [0.083-0.760] | 2.438 | 0.015 | 1.810 | 0.405 | 0.000 |  |
| **IL-4** | All | 10 | 362 | 308 | RE | 0.203 [-0.091-0.497] | 1.354 | 0.176 | 30.293 | <0.001 | 70.290 | 0.337 |
|  | Plasma | 7 | 276 | 252 | RE | 0.097 [-0.188-0.382] | 0.665 | 0.506 | 15.131 | 0.019 | 60.345 |  |
|  |  | 3 | 86 | 56 | RE | 0.454[-0.278-1.187] | 1.216 | 0.224 | 8.232 | 0.016 | 75.706 |  |
| **RANTES** | All | 8 | 287 | 239 | RE | 0.279 [-0.166-0.724] | 1.228 | 0.220 | 41.490 | <0.001 | 83.128 | 0.724 |
|  | Plasma | 5 | 232 | 203 | RE | 0.344 [-0.234-0.922] | 1.166 | 0.244 | 34.331 | <0.001 | 88.349 |  |
|  | Serum | 3 | 55 | 36 | RE | 0.145 [-0.615-0.905] | 0.375 | 0.708 | 6.452 | 0.040 | 69.004 |  |
| **IL-18** | All | 4 | 147 | 117 | RE | -0.260 [-1.328-0.808] | -0.478 | 0.633 | 49.952 | <0.001 | 93.994 | 0.306 |
|  | Serum | 3 | 119 | 89 | RE | -0.370 [-1.895-1.155] | -0.476 | 0.634 | 49.637 | <0.001 | 95.971 |  |
| **IL-12** | All | 6 | 181 | 145 | RE | 1.047 [-0.081-2.176] | 1.820 | 0.069 | 94.222 | <0.001 | 94.693 | 0.544 |
|  | Plasma | 3 | 96 | 92 | RE | 1.763 [0.431-3.095] | 2.593 | 0.010 | 28.004 | <0.001 | 92.858 |  |
|  | Serum | 3 | 85 | 53 | RE | 0.293 [-1.047-1.632] | 0.428 | 0.669 | 18.826 | <0.001 | 89.376 |  |
| **IL-12p40** | All | 6 | 224 | 212 | RE | 0.466 [-0.104-1.037] | 1.602 | 0.109 | 38.208 | <0.001 | 86.914 | 0.372 |
|  | Plasma | 4 | 167 | 155 | RE | 0.364 [-0.005-0.733] | 1.931 | 0.053 | 6.710 | 0.082 | 55.288 |  |
| **IL-12p70** | All | 5 | 151 | 143 | RE | 0.939 [0.090-1.788] | 2.167 | 0.030 | 43.601 | <0.001 | 90.826 | 0.313 |
|  | Plasma | 4 | 130 | 128 | RE | 1.224 [0.409-2.038] | 2.944 | 0.003 | 25.264 | <0.001 | 88.125 |  |
| **IP-10** | All | 6 | 208 | 160 | RE | -0.447 [-1.020-0.126] | -1.528 | 0.127 | 31.586 | <0.001 | 84.170 | 0.027 |
|  | Plasma | 3 | 153 | 124 | RE | -0.088 [-0.572-0.397] | -0.354 | 0.723 | 7.796 | 0.020 | 74.346 |  |
|  | Serum | 3 | 55 | 36 | RE | -1.111 [-2.612-0.391] | -1.449 | 0.147 | 19.859 | <0.001 | 89.929 |  |
| **Mig** | All | 4 | 120 | 104 | RE | -0.520 [-1.136-0.096] | -1.654 | 0.098 | 14.991 | 0.002 | 79.988 | 0.433 |
|  | Plasma | 3 | 98 | 91 | FE | -0.260 [-0.545-0.025] | -1.787 | 0.074 | 4.343 | 0.114 | 53.948 |  |
| **IL-5** | All | 6 | 237 | 215 | RE | 0.273 [-0.095-0.640] | 1.452 | 0.146 | 16.856 | 0.005 | 70.336 | 0.233 |
|  | Plasma | 4 | 195 | 178 | RE | 0.125 [-0.286-0.535] | 0.595 | 0.552 | 10.620 | 0.014 | 71.751 |  |
| **IL-7** | All | 6 | 157 | 143 | FE | 0.262 [0.036-0.488] | 2.272 | 0.023 | 8.064 | 0.153 | 37.994 | 0.315 |
|  | Plasma | 4 | 115 | 106 | FE | 0.210 [-0.053-0.472] | 1.566 | 0.117 | 3.945 | 0.267 | 23.962 |  |
| **IL-13** | All | 8 | 363 | 262 | RE | -0.065 [-0.499-0.369] | -0.295 | 0.768 | 44.399 | <0.001 | 84.212 | 0.953 |
|  | Plasma | 4 | 195 | 178 | RE | -0.011 [-0.564-0.541] | -0.040 | 0.968 | 19.002 | <0.001 | 84.213 |  |
|  | Serum | 4 | 168 | 84 | RE | -0.137[-0.964-0.691] | -0.324 | 0.746 | 25.148 | <0.001 | 88.071 |  |
| **IL-23** | All | 3 | 102 | 73 | RE | -0.448 [-1.062-0.166] | -1.431 | 0.152 | 5.684 | 0.058 | 64.813 | 0.023 |
| **G-CSF** | All | 4 | 164 | 101 | RE | -0.101 [-0.642-0.439] | -0.367 | 0.713 | 11.796 | 0.008 | 74.567 | 0.719 |
|  | Plasma | 3 | 152 | 93 | RE | -0.030 [-0.691-0.631] | -0.088 | 0.930 | 11.463 | 0.003 | 82.553 |  |
| **GM-CSF** | All | 4 | 179 | 158 | FE | 0.092 [-0.120-0.305] | 0.849 | 0.396 | 2.300 | 0.513 | 0.000 | 0.676 |
|  | Serum | 3 | 167 | 150 | FE | 0.083 [-0.136-0.302] | 0.742 | 0.458 | 2.192 | 0.334 | 8.763 |  |
| **PDGF-BB** | All | 4 | 96 | 92 | RE | 0.261 [-0.163-0.686] | 1.208 | 0.227 | 6.236 | 0.101 | 51.894 | 0.090 |
| **IL-2R** | All | 4 | 91 | 94 | RE | 0.235 [-0.516-0.985] | 0.613 | 0.540 | 17.672 | 0.001 | 83.024 | 0.311 |
| **IL-9** | All | 3 | 120 | 122 | FE | 0.153 [-0.098-0.404] | 1.192 | 0.233 | 2.514 | 0.285 | 20.439 | 0.295 |

RE, random-effects model; FE, fixed-effects model.
