# Supplementary material for: Association of Peripheral Blood Levels of Cytokines With Autism Spectrum Disorder: A Meta-Analysis
Source: Front Psychiatry. 2021 Jul 2;12:670200. doi: 10.3389/fpsyt.2021.670200 (PMC8283413; doi:10.3389/fpsyt.2021.670200)
Supplement: Supplementary file 3 [file Data_Sheet_1.doc]

**Supplemental Information**

**Supplementary Figure 1.** Meta-regression (IL-6).


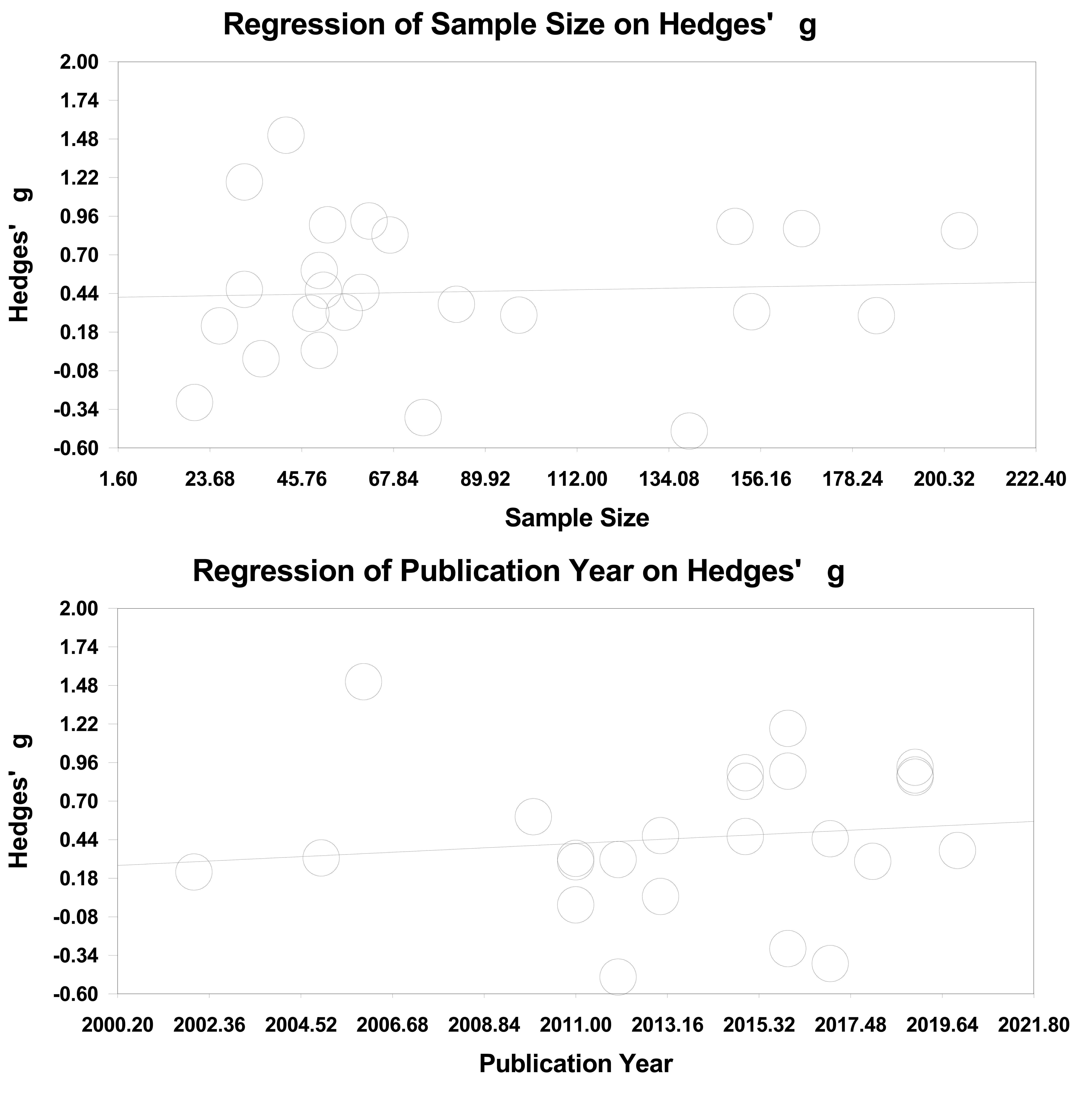


**Supplementary Figure 2.** Meta-regression (IFNγ).


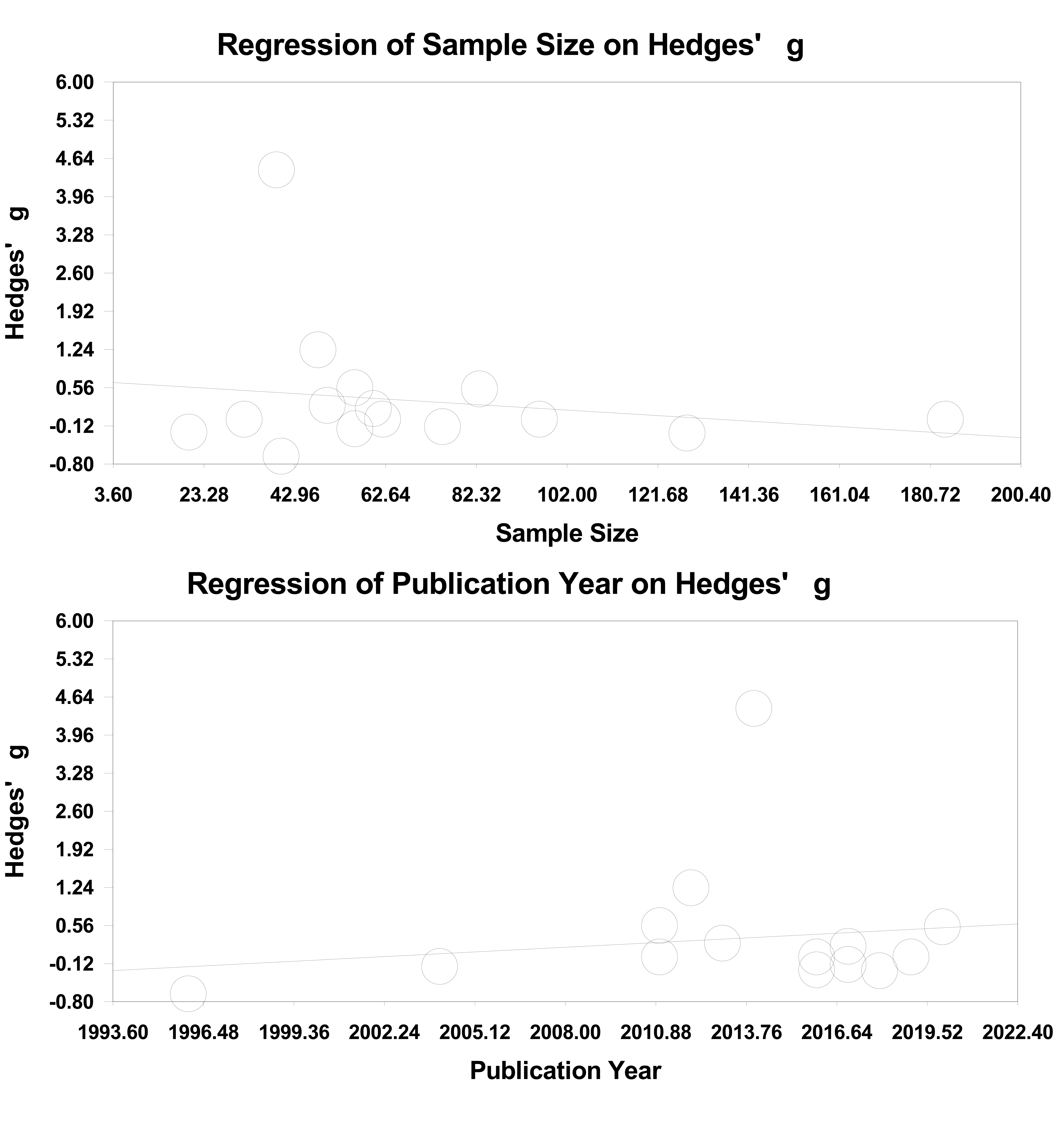


**Supplementary Figure 3.** Meta-regression (IL-8).


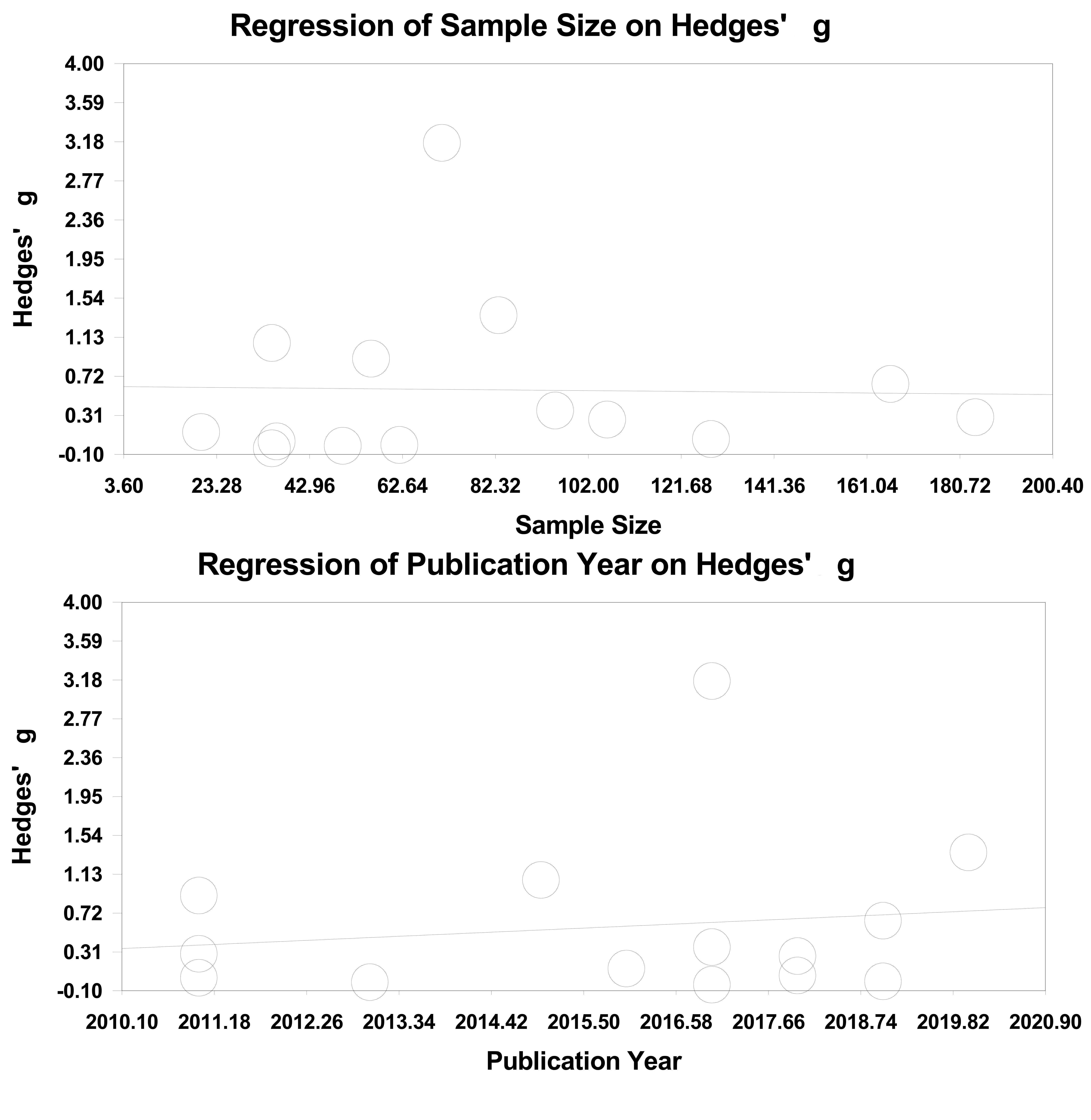


**Supplementary Figure 4.** Meta-regression (TNF-α).

**
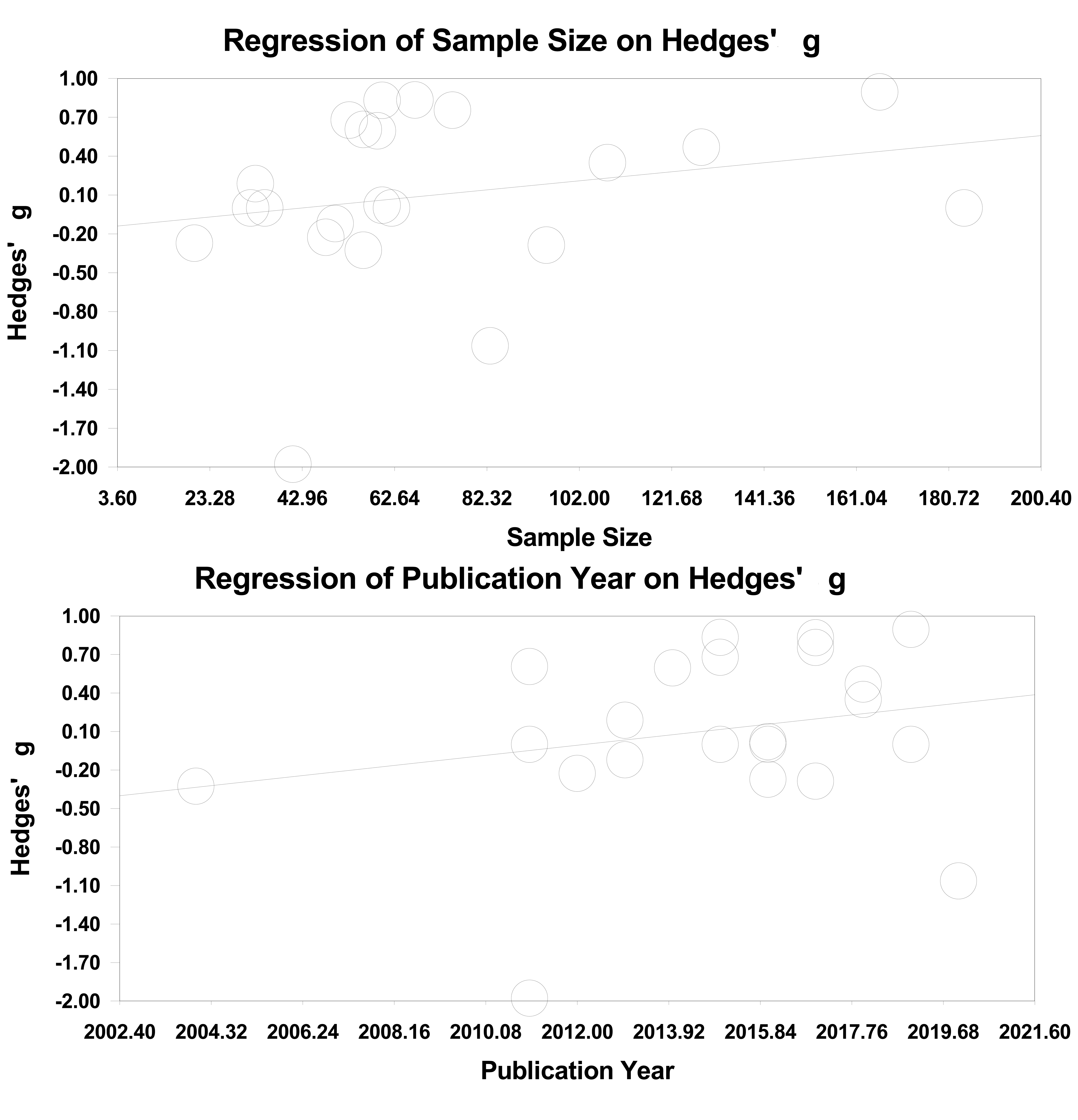
**

**Supplementary Figure 5.** Meta-regression (IL-1β).

**
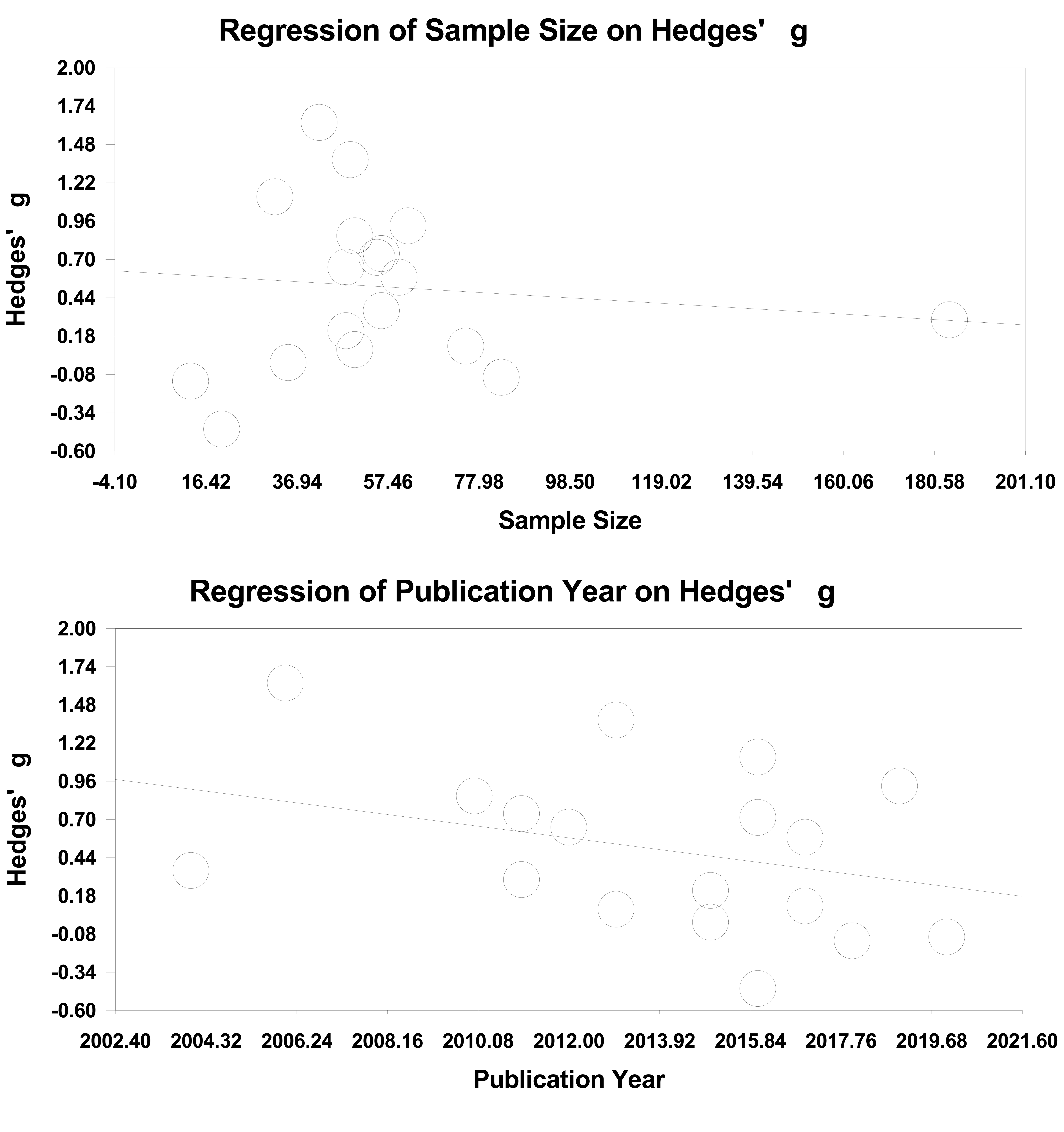
**

**Supplementary Figure 6.** Meta-regression (IL-10).

**
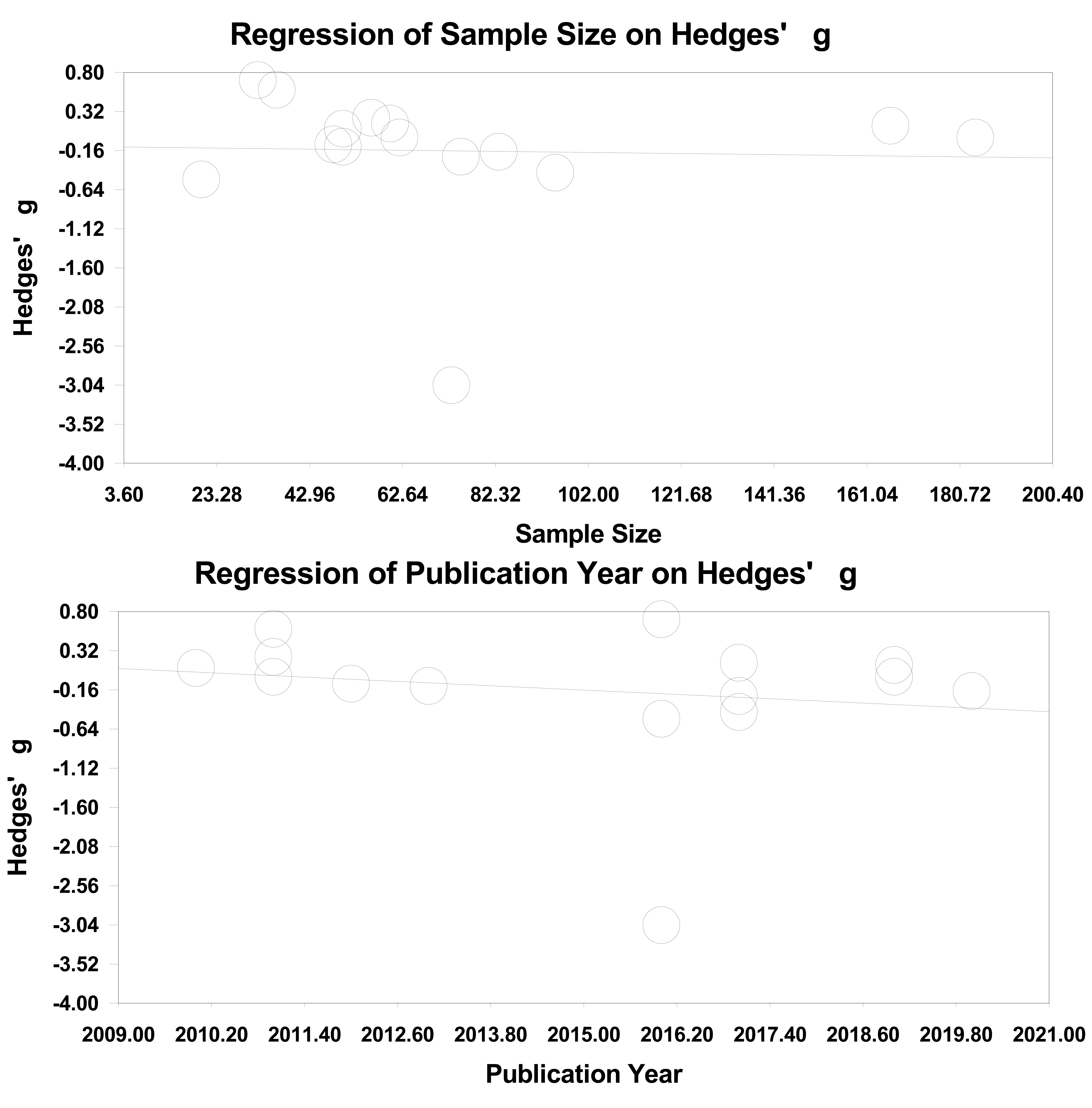
**

**Supplementary Figure 7.** Meta-regression (IL-17).

**
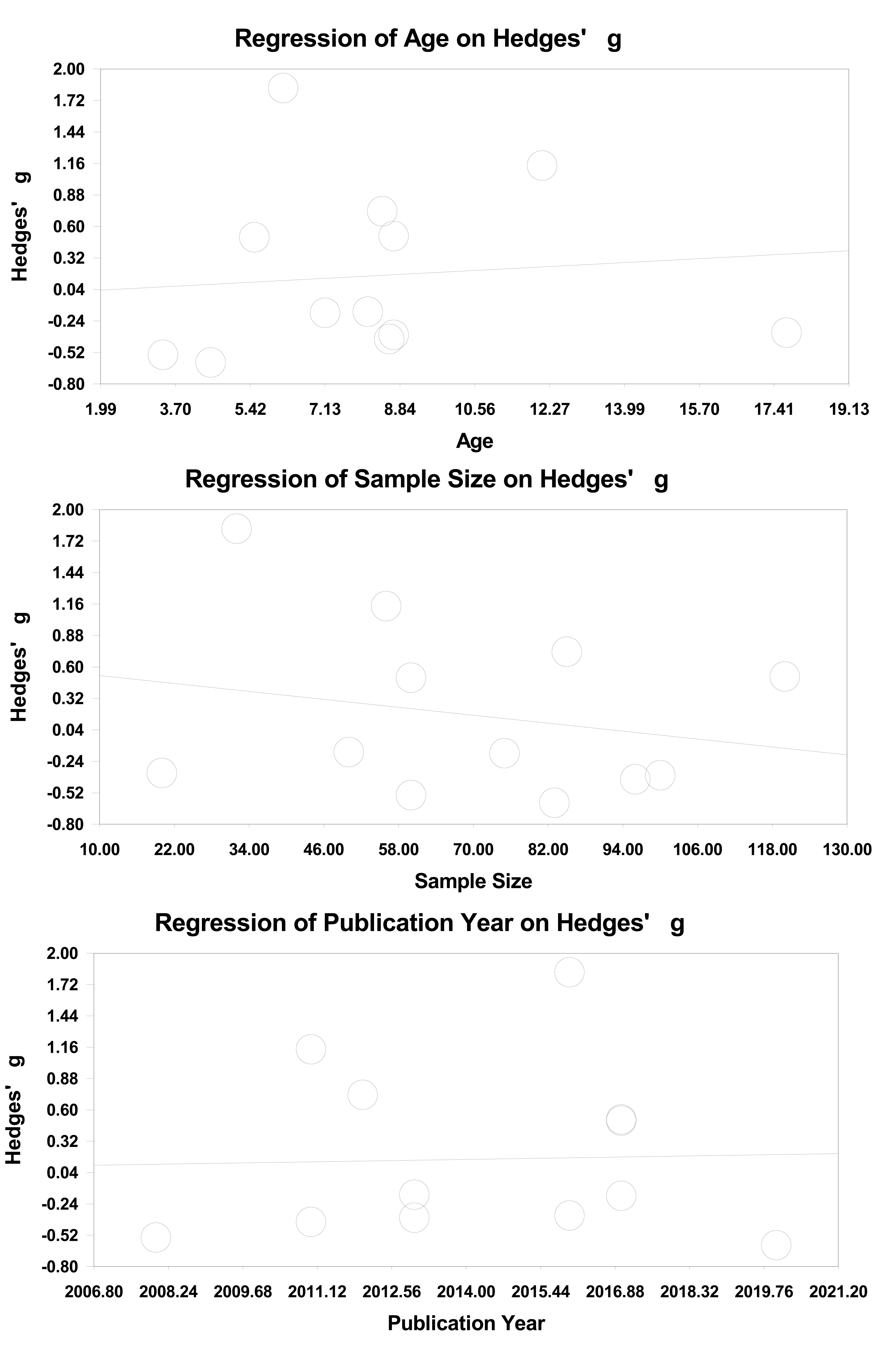
**

**Supplementary Figure 8.** Meta-regression (IL-4).

**
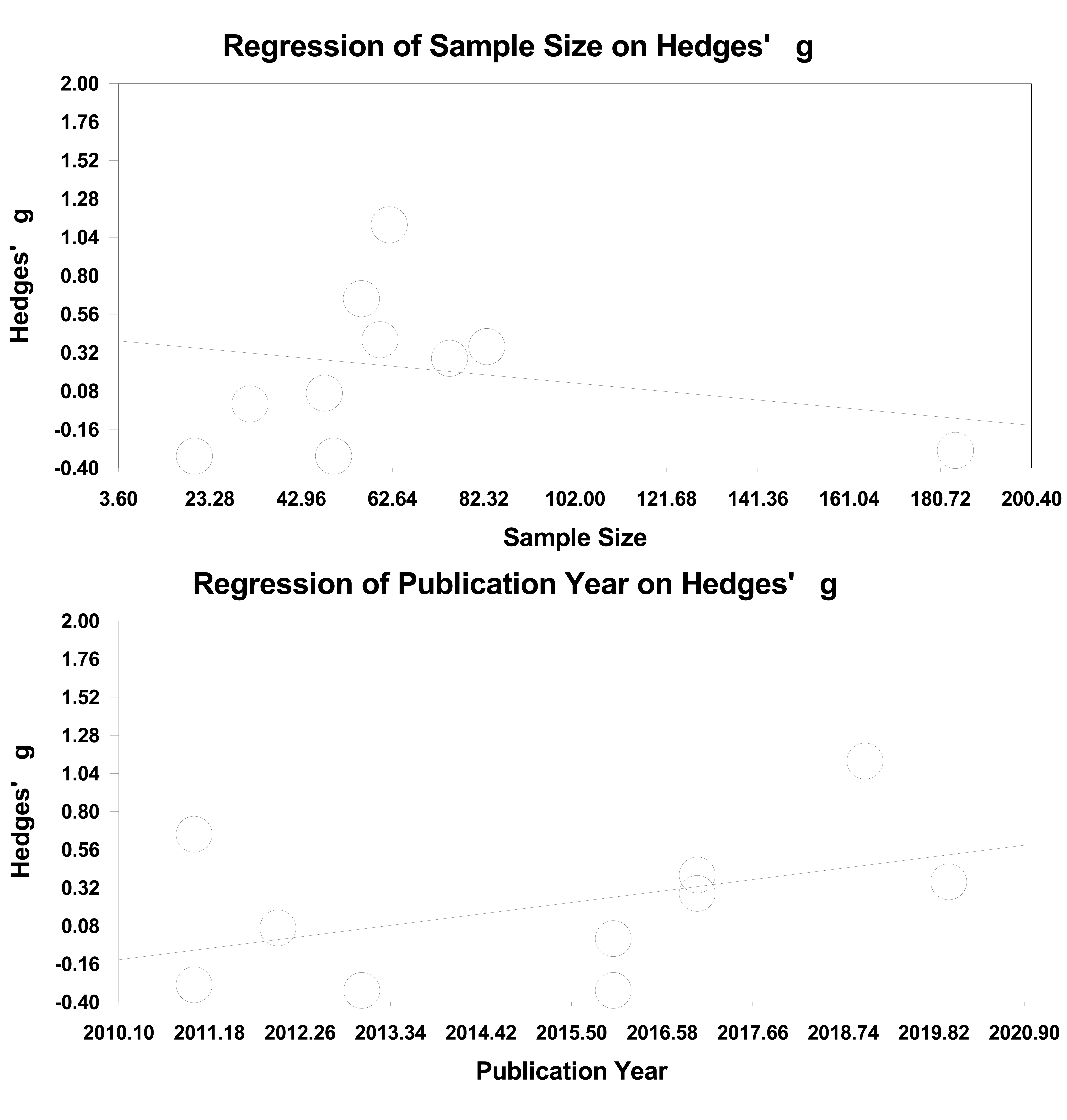
**

**Supplementary Figure 9.** Galbraith plot for the random-effect meta-analysis (IL-6).

**
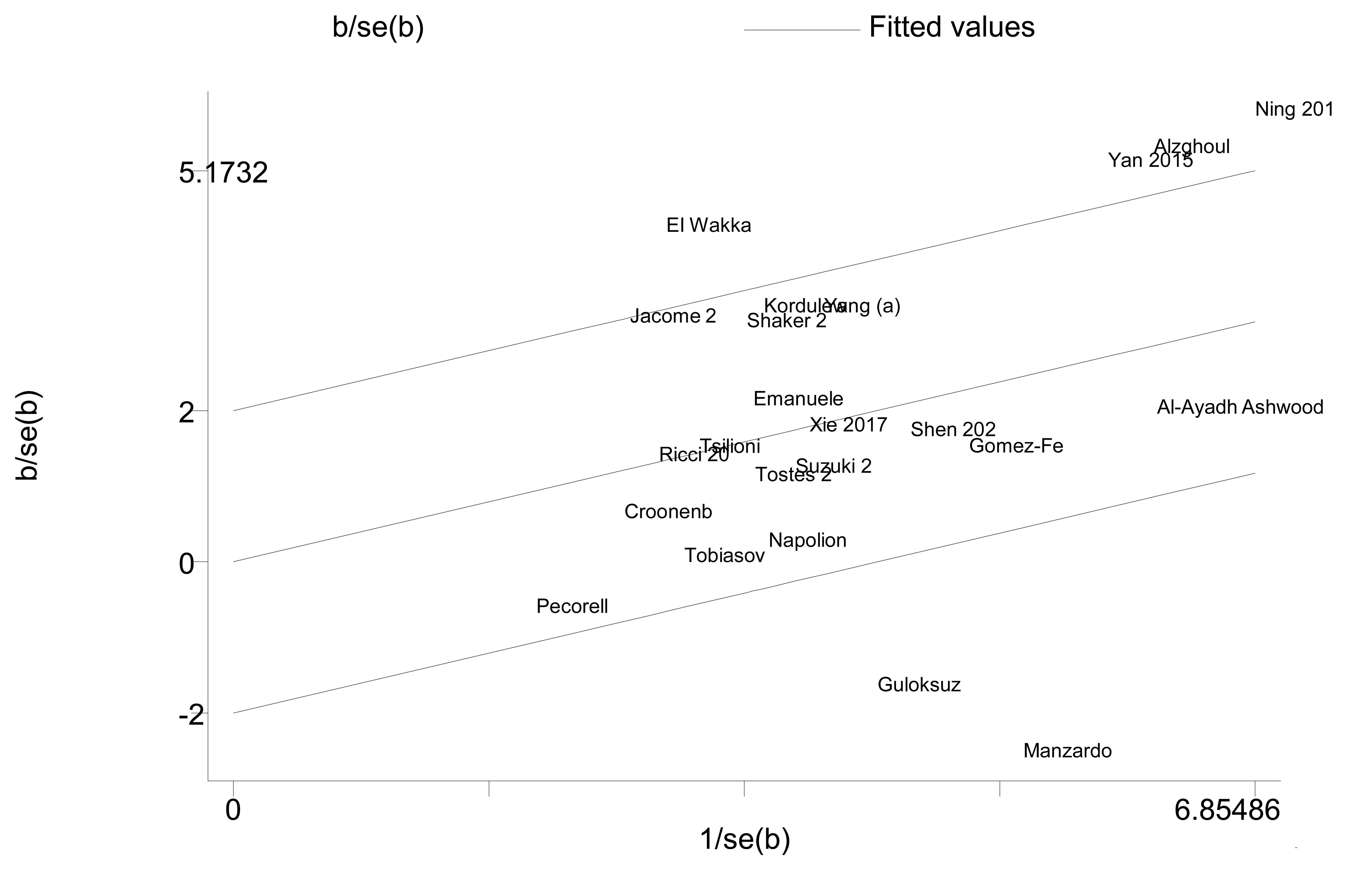
**

**Supplementary Figure 10.** Galbraith plot for the random-effect meta-analysis (IFNγ).

**
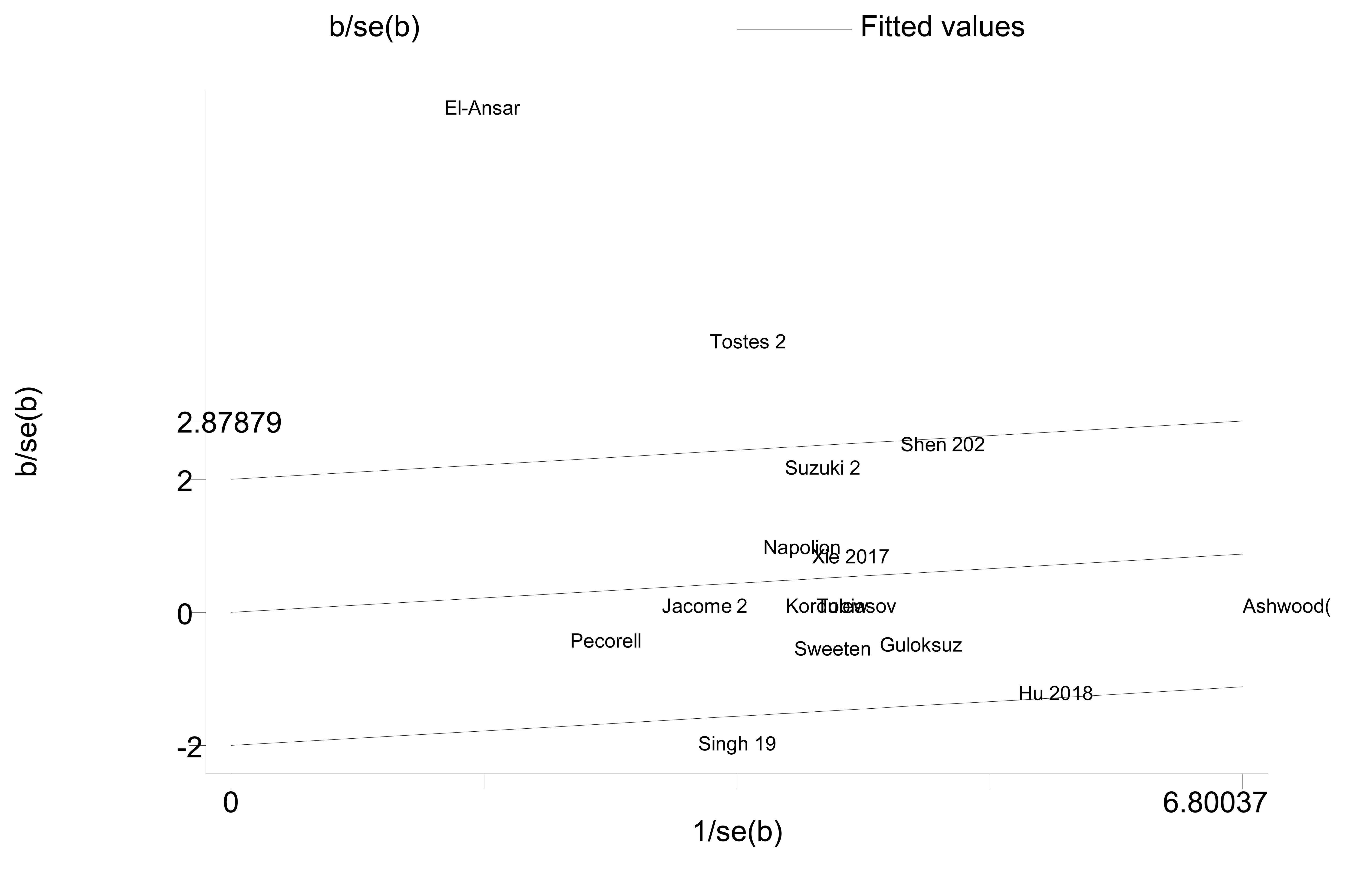
**

**Supplementary Figure 11.** Galbraith plot for the random-effect meta-analysis (IL-8).

**
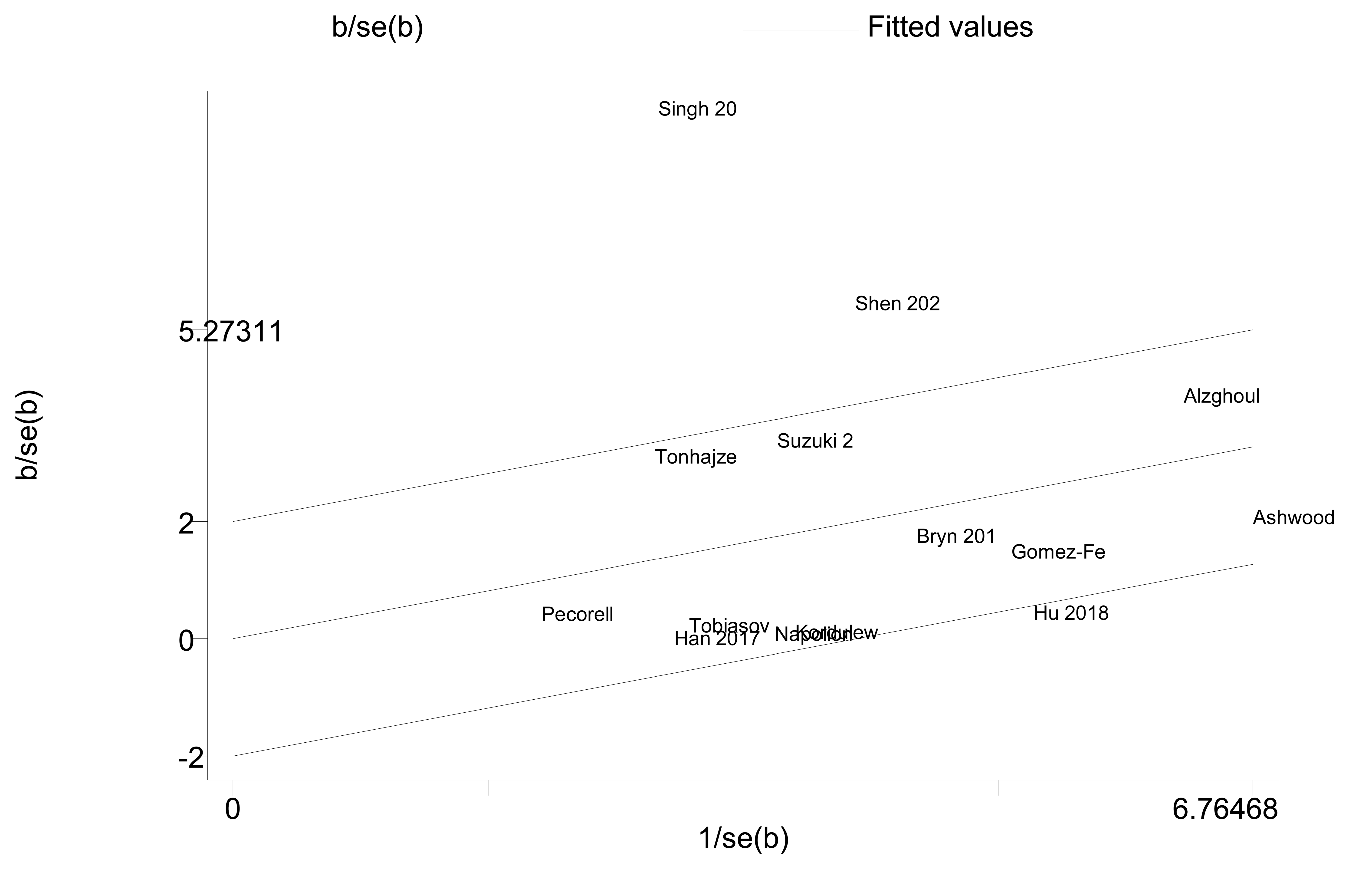
**

**Supplementary Figure 12.** Galbraith plot for the random-effect meta-analysis (TNF-α).

**
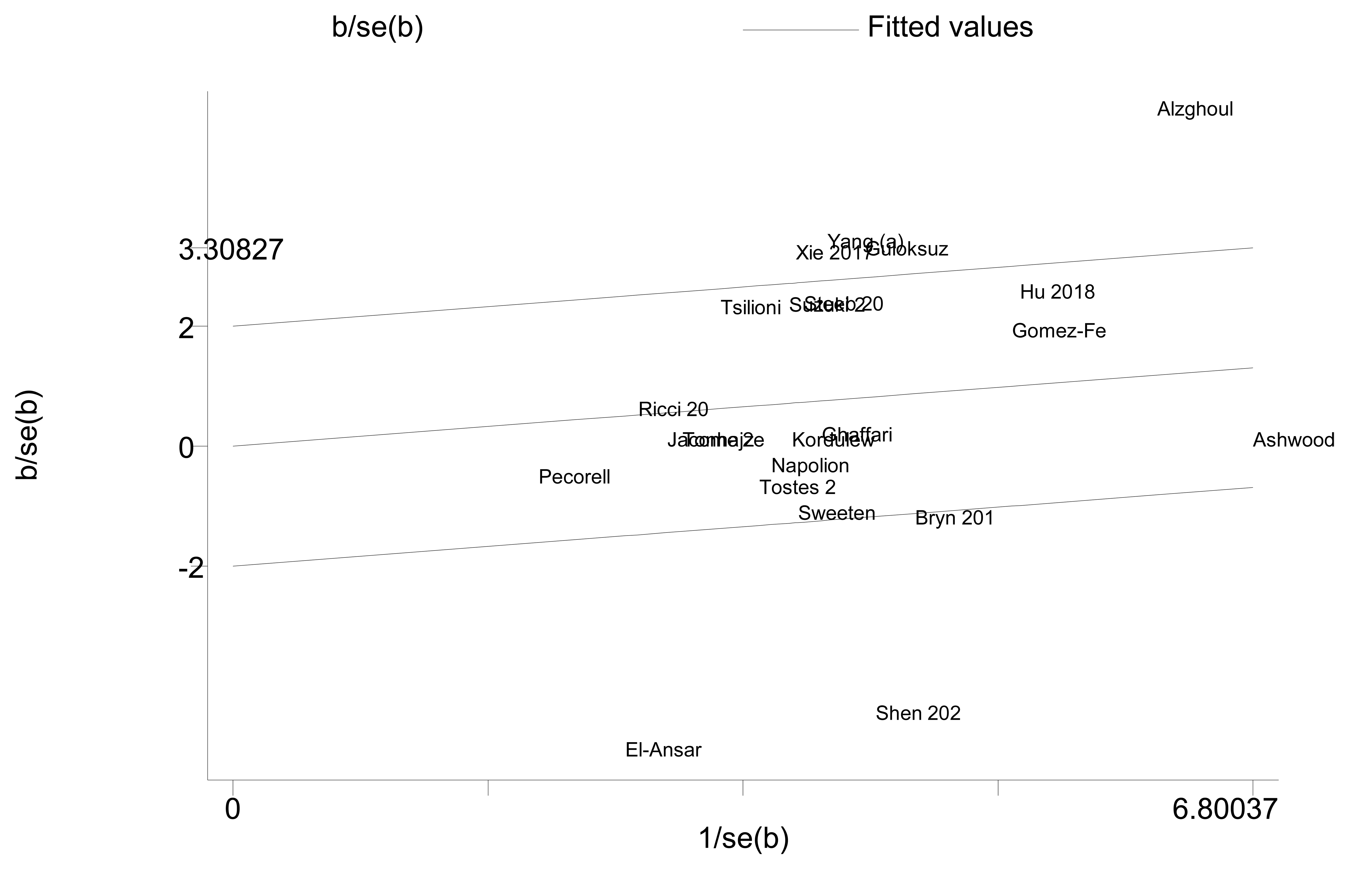
**

**Supplementary Figure 13.** Galbraith plot for the random-effect meta-analysis (IL-1β).

**
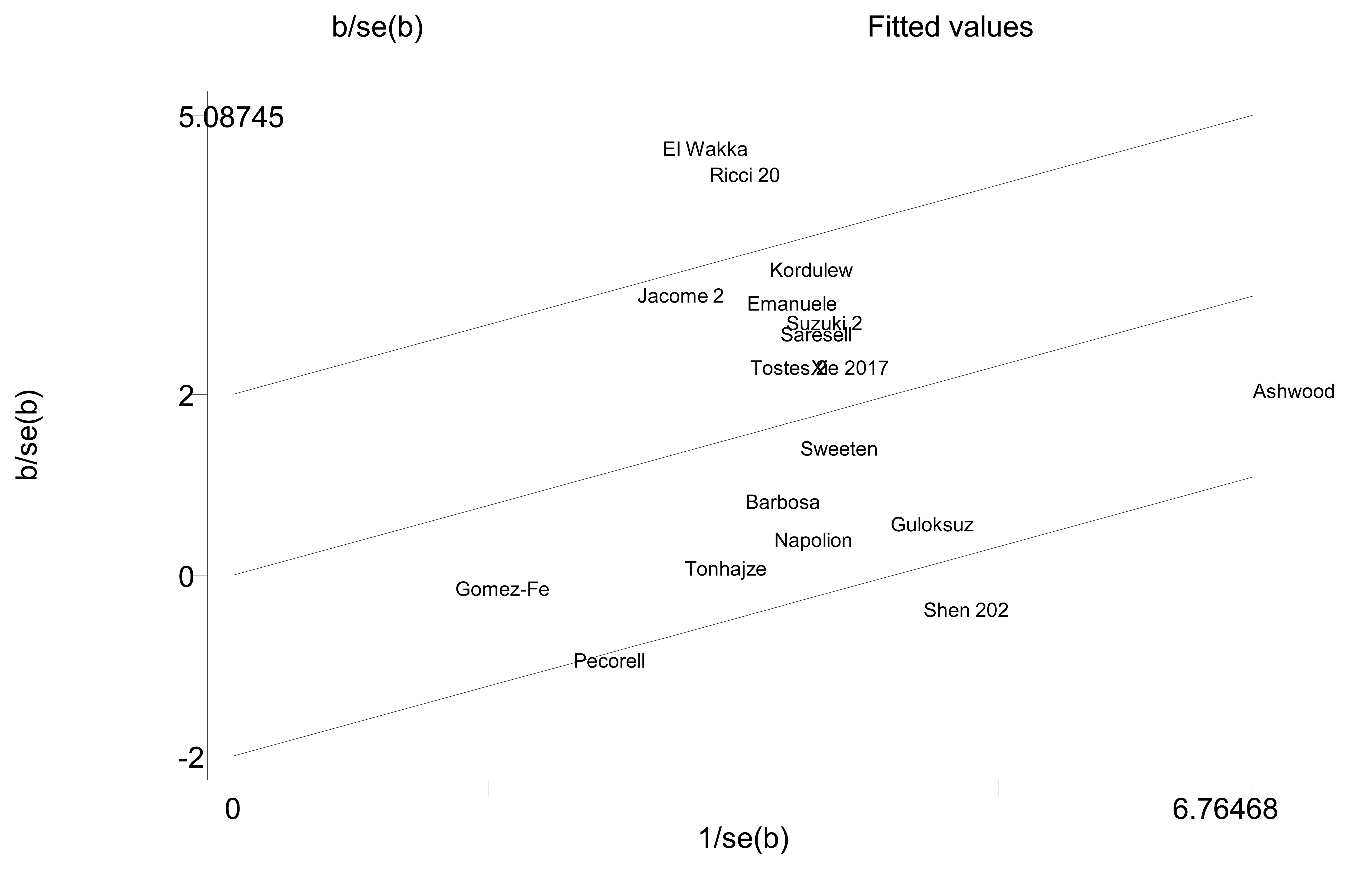
**

**Supplementary Figure 14.** Galbraith plot for the random-effect meta-analysis (IL-10).

**
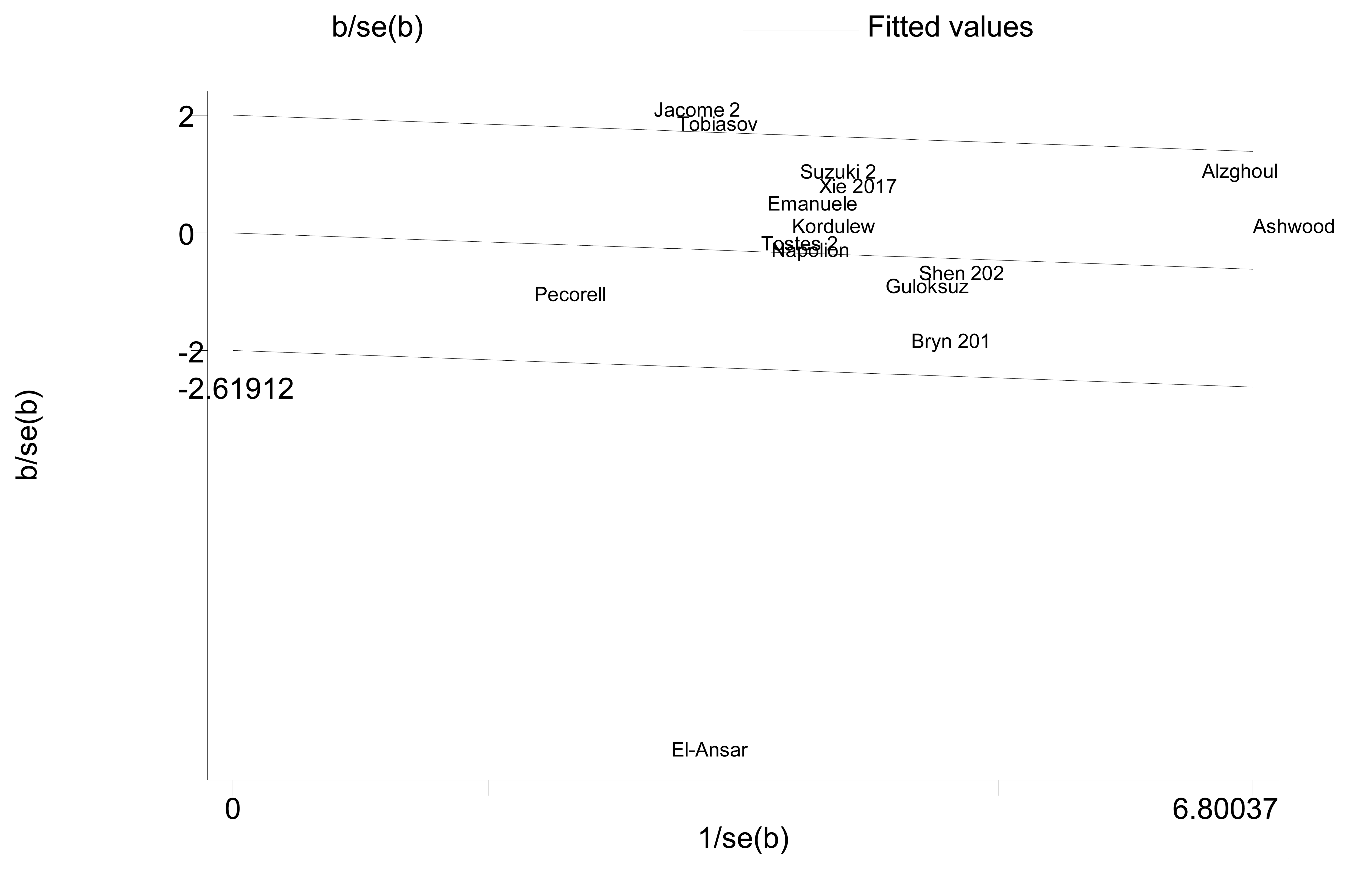
**

**Supplementary Figure 15.** Galbraith plot for the random-effect meta-analysis (IL-17).

**
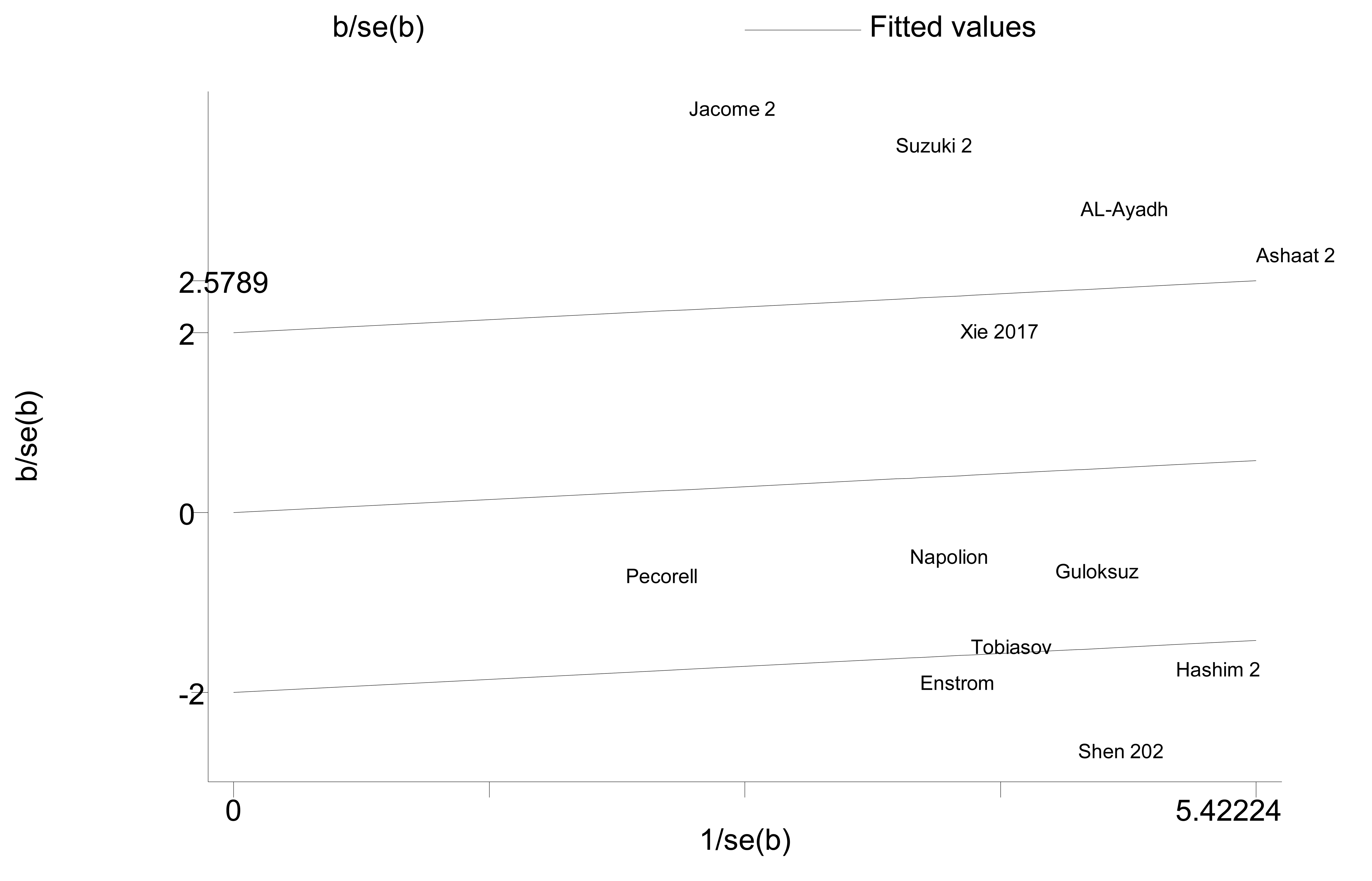
**

**Supplementary Figure 16.** Galbraith plot for the random-effect meta-analysis (IL-4).


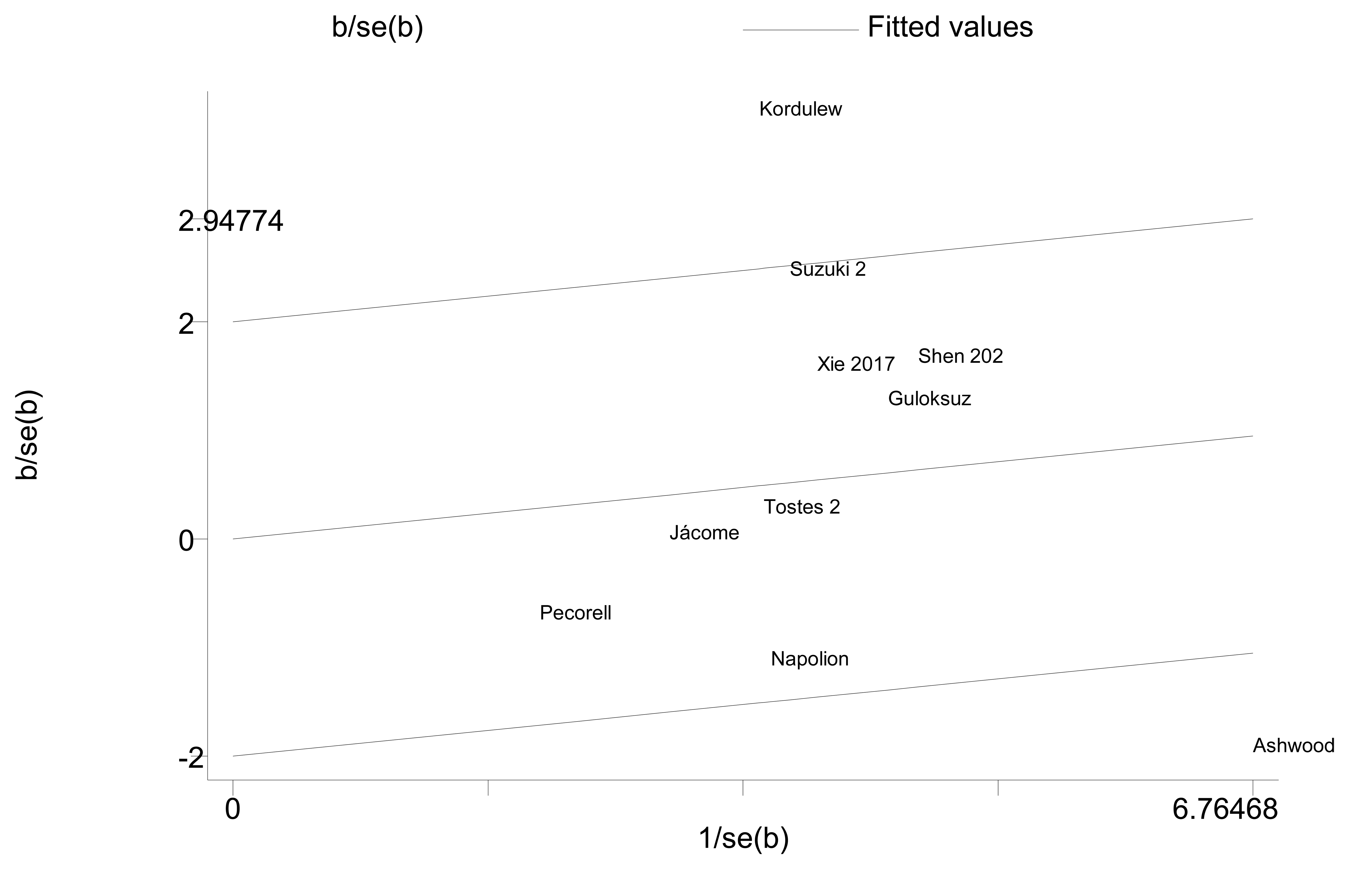


**Supplementary Figure 17.** Sensitivity analysis for IL-4.

**
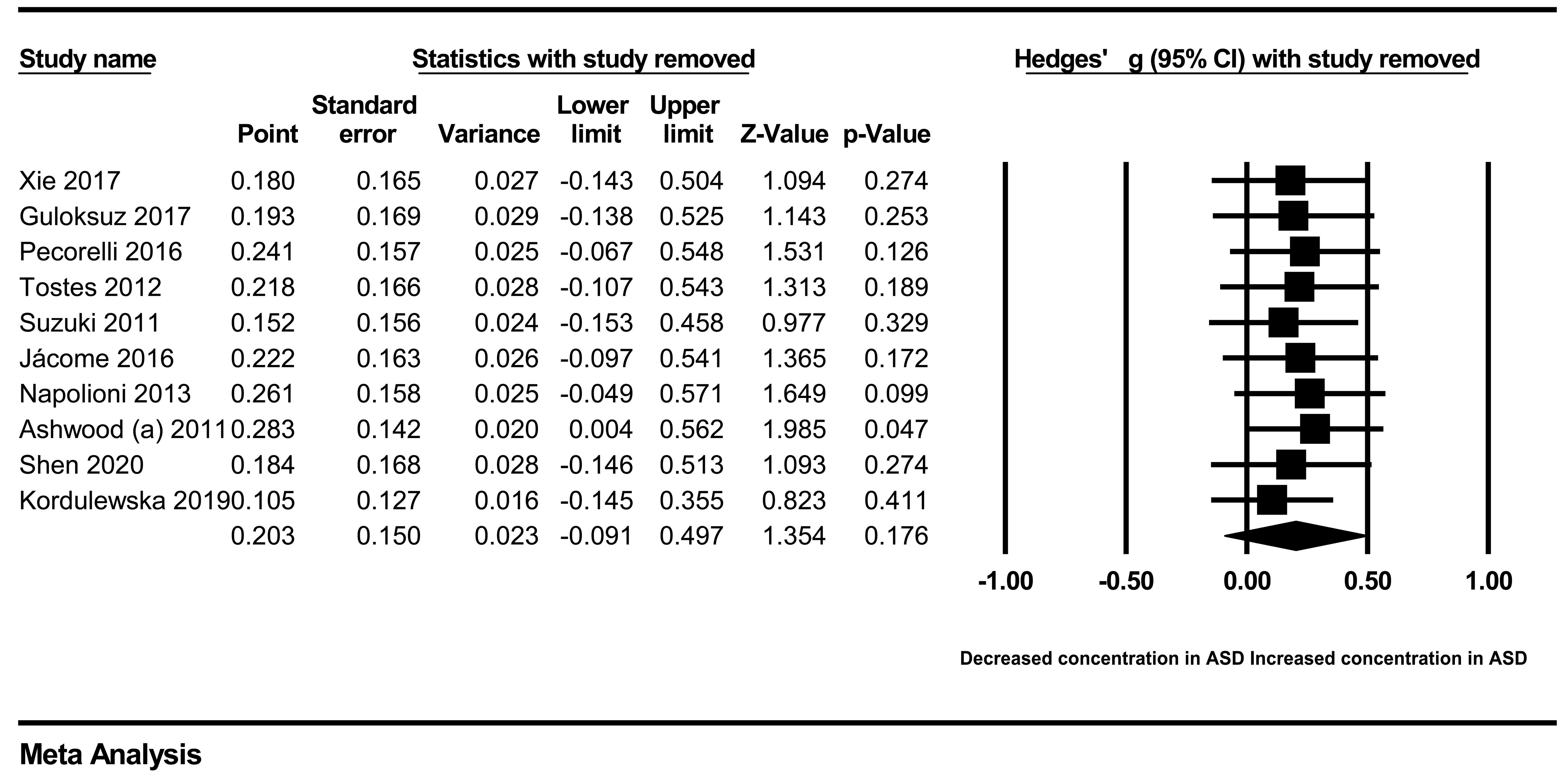
**
